# Activated Protein C Ameliorates Diabetic Cardiomyopathy *via* Modulating OTUB1/YB-1/MEF2B Axis

**DOI:** 10.3389/fcvm.2021.758158

**Published:** 2021-10-29

**Authors:** Xiaodan Zhong, Tao Wang, Yang Xie, Mengwen Wang, Wenjun Zhang, Lei Dai, Jinsheng Lai, Xiang Nie, Xingwei He, Thati Madhusudhan, Hesong Zeng, Hongjie Wang

**Affiliations:** ^1^Division of Cardiology, Department of Internal Medicine, Tongji Hospital, Tongji Medical College, Huazhong University of Science and Technology, Wuhan, China; ^2^Hubei Key Laboratory of Genetics and Molecular Mechanisms of Cardiological Disorders, Wuhan, China; ^3^Department of Cardiology, Affiliated Hospital of Weifang Medical University, Weifang, China; ^4^Center for Thrombosis and Hemostasis, University Medical Center Mainz, Mainz, Germany

**Keywords:** diabetic cardiomyopathy, activated protein C, OTUB1, YB-1, MEF2B

## Abstract

**Aims:** The pathogenesis of diabetic cardiomyopathy (DCM) is complex and the detailed mechanism remains unclear. Coagulation protease activated Protein C (aPC) has been reported to have a protective effect in diabetic microvascular disease. Here, we investigated whether aPC could play a protective role in the occurrence and development of major diabetic complication DCM, and its underlying molecular mechanism.

**Methods and Results:** In a mouse model of streptozotocin (STZ) induced DCM, endogenous aPC levels were reduced. Restoring aPC levels by exogenous administration of zymogen protein C (PC) improved cardiac function of diabetic mice measured by echocardiography and invasive hemodynamics. The cytoprotective effect of aPC in DCM is mediated by transcription factor Y-box binding protein-1 (YB-1). Mechanistically, MEF2B lies downstream of YB-1 and YB-1/MEF2B interaction restrains deleterious MEF2B promoter activity in DCM. The regulation of YB-1 on MEF2B transcription was analyzed by dual-luciferase and chromatin immunoprecipitation assays. In diabetic mice, aPC ameliorated YB-1 degradation *via* reducing its K48 ubiquitination through deubiquitinating enzyme otubain-1 (OTUB1) and improving the interaction between YB-1 and OTUB1. Using specific agonists and blocking antibodies, PAR1 and EPCR were identified as crucial receptors for aPC's dependent cytoprotective signaling.

**Conclusion:** These data identify that the cytoprotective aPC signaling *via* PAR1/EPCR maintains YB-1 levels by preventing the ubiquitination and subsequent proteasomal degradation of YB-1 *via* OTUB1. By suppressing MEF2B transcription, YB-1 can protect against DCM. Collectively, the current study uncovered the important role of OTUB1/YB-1/MEF2B axis in DCM and targeting this pathway might offer a new therapeutic strategy for DCM.

**Translational Perspective:** DCM is emerging at epidemic rate recently and the underlying mechanism remains unclear. This study explored the protective cell signaling mechanisms of aPC in mouse models of DCM. As a former FDA approved anti-sepsis drug, aPC along with its derivatives can be applied from bench to bed and can be explored as a new strategy for personalized treatment for DCM. Mechanistically, OTUB1/YB-1/MEF2B axis plays a critical role in the occurrence and development of DCM and offers a potential avenue for therapeutic targeting of DCM.

## Graphical Abstract

In mouse model of chronic diabetes mellitus, persistent hyperglycemia impaired thrombin-thrombomodulin-EPCR dependent PC activation. The reduced aPC-dependent cytoprotective signaling *via* PAR1/EPCR supressed OTUB1 expression resulting in augmented K48 ubiquitination and proteasomal degradation of the transcription factor YB-1. Within the nucleus, YB-1 binds to MEF2B promoter and restrains its transcription. Accordingly, ubiquitination and reduced protein levels of YB-1 compromised its inhibitory effect on MEF2B promoter and enhanced MEF2B mRNA transcription. Subsequently, elevated MEF2B expression disrupted the homeostasis of cardiomyocytes, rendering them susceptible to DCM. Exogenous administration of PC restores OTUB1/YB-1/MEF2B dependent cytoprotective responses and ameliorates development of DCM. 
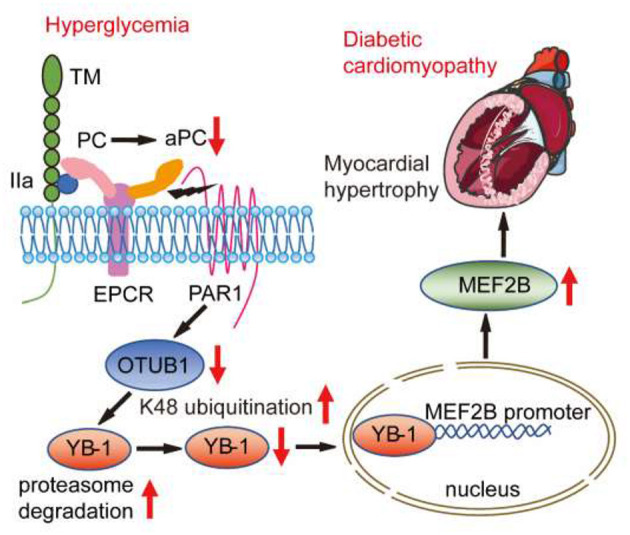

## Introduction

Diabetic cardiomyopathy (DCM), which is defined as abnormal myocardial structure and function without other cardiac risk factors, is increasing at epidemic rate and poses serious threats to human health (1). Many potential mechanisms have been identified in recent years, including systemic metabolic disorder, mitochondrial damage, oxidative stress, microcirculation disorder, inflammation, and heart remodeling, but the precise mechanism remains unclear (2–4). Moreover, traditional heart failure therapy or blood glucose control cannot reverse the progression of DCM (5), making it a major unmet clinical need among cardiovascular diseases.

Activated protein C (aPC), the active form of the zymogen protein C (PC), is known to have a strong protective effect in pre-clinical models of diabetes mellitus (6). It has been reported that the plasma aPC levels were significantly reduced in diabetic mice (7). Genetic or therapeutic supplement of aPC can prevent cell damage and progressive cell loss in type 1 diabetic mice and ameliorate inflammation and autoimmunity in the pancreatic islets (8). In pre-clinical studies, loss of aPC generation impaired the renal epithelial-podocyte function and ultimately led to glomerular filtration barrier dysfunction and diabetic nephropathy (DN), while exogenous administration of aPC effectively alleviated it (9). Although aPC has been reported to have a protective effect in diabetes mellitus (DM) and associated microvascular complications of the kidney, the role of aPC in other major macrovascular complications of DM, such as DCM remains unknown. The cytoprotective effects of aPC are mediated *via* cell-specific receptor complexes (10). In endothelial cells, the cytoprotective signaling of aPC is predominately regulated *via* endothelial protein C receptor (EPCR) dependent activation of protease activated receptor-1 (PAR1). However, in specialized renal epithelial podocytes, we have recently identified that aPC-dependent cytoprotective effects are independent of EPCR and requires integrin α_V_β3 and protease activated receptor-3 (PAR3) (11–13). However, the cell-specific receptor complexes and the signaling mechanisms through which aPC confers protective effects in cardiomyocytes remain to be explored.

Our recent data shows that aPC can influence the ubiquitination of Y Box binding protein-1 (YB-1) by regulating the expression of deubiquitinating enzyme otubain-1 (OTUB1), thus playing a protective role in mouse model of renal ischemia reperfusion injury (14). YB-1 is a member of cold shock superfamily, which can influence the long-term prognosis of heart transplant patients and the prognosis of myocardial infarction in mice (15, 16). Since YB-1 could be regulated by aPC, we presumed that YB-1 could be a potential intracellular target in the regulation of DCM. Ubiquitination is a common post-translational modification that modulates protein stability and/or degradation, which is mechanistically linked to multiple processes of translation (17). Protein stability is critically regulated by the enzymes that modulate ubiquitination and deubiquitination. Deubiquitinating enzyme OTUB1 can regulate the ubiquitination and degradation of proteins, affecting cell metabolism, differentiation, proliferation, and apoptosis (18). We recently demonstrated that OTUB1 could maintain the protein stability of YB-1 in renal ischemia-reperfusion injury (14). However, the mechanisms through which OTUB1 modulates YB-1 expression and confers the cytoprotective responses remain unclear. Here, in mouse models of DCM, we uncovered a novel function of OTUB1/YB-1 axis in regulation of the MEF2B transcription which was causatively linked to DCM. Additionally, we explored whether restoring aPC levels *in vivo* by pharmacological administration of zymogen PC maintained cytoprotective OTUB1/YB-1/MEF2B pathway in DCM.

## Methods

### Experimental Diabetic Mouse Model

All animal procedures were carried out according to the protocols approved by the Institutional Animal Research Committee of Tongji Medical College, complied with standards stated in the National Institutes of Health Guidelines for the Care and Use of Laboratory Animals and the Chinese Academy of Sciences, in accordance to the ARRIVE guidelines (19) and complied with the principles of replacement, refinement, and reduction (the 3Rs). Six-week-old C57BL/6J male mice were purchased from Beijing Vital River Laboratory Animal Technology Co., Ltd. The weight of animal was around 23 g. Animals were housed at the specific pathogen free animal care facility of Tongji Medical College with a 12 h dark-light cycle and allowed free access to water and food, while water and food were refreshed every 3 days. The room temperature was 23 ± 1°C and the humidity was around 50%. Type 1 diabetes mellitus model was induced by 5 continuous intraperitoneal injection of streptozotocin (STZ). Continuous inhalation anesthesia with 0.5% isoflurane was employed for echocardiography and intraperitoneal injection with pentobarbitone sodium at the dose of 80 mg/kg was used for hemodynamic measurements. After the Millar catheter measurement, mice were received an excessive pentobarbitone sodium solution intraperitoneal injection for euthanasia. For further information, see the Supplementary Material.

### Echocardiography and Hemodynamic Measurements

Echocardiography was performed by ultrasound professionals with a VisualSonics vevo 770 imaging system (VisualSonics, Toronto, Canada). For further information, see the Supplementary Material.

### Histology, Immunohistochemistry, and Immunofluorescence

To evaluate the cell surface area of cardiomyocyte, hematoxylin-eosin (HE) staining and wheat germ agglutinin (WGA) staining were performed. Immunohistochemistry was employed to detect the expression and distribution of YB-1 and OTUB1 and immunofluorescence was used to identify the expression of EPCR in H9c2 cells. For further information, see the Supplementary Material.

### ELISA

Plasma aPC levels were determined by mouse or human activated protein C ELISA Kit (CUSABIO). Plasma insulin, triglyceride and total cholesterol levels were detected with Insulin assay kit, Triglyceride assay kit and Total Cholesterol assay kit (Nanjing Jiancheng Bioengineering Institute). For further information, see the Supplementary Material.

### Immunoprecipitation and Immunoblotting

For further information, see the Supplementary Material.

### Cell Culture and Treatment

The H9c2 cell line and 293T cell line were purchased from the American Type Culture Collection (ATCC). Manipulation was described in the Supplementary Material.

### Generation of Knockdown Cell Lines

For further information, see the Supplementary Material.

### Quantitative Real-Time PCR

Total RNA of mice heart tissues was extracted with Trizol reagent as described previously. For further information, see the Supplementary Material.

### Chromatin Immunoprecipitation Assay

H9c2 cells were used for ChIP assay. For further information, see the Supplementary Material.

### Dual-Luciferase Assay

For further information, see the Supplementary Material.

### Reporter Plasmid Construction

For further information, see the Supplementary Material.

### Statistical Analysis

All data were summarized as mean ± SD. Statistical analysis was performed with SPSS 24.0 software. Differences between two groups were evaluated by unpaired Student's *t*-test. Comparisons between three or more groups were made with one-way ANOVA or two-way ANOVA with Bonferroni post-test. *P* < 0.05 was accepted as statistical significance.

## Results

### Activated PC Maintains YB-1 Expression in Diabetic Cardiomyopathy

**Figure 1:**
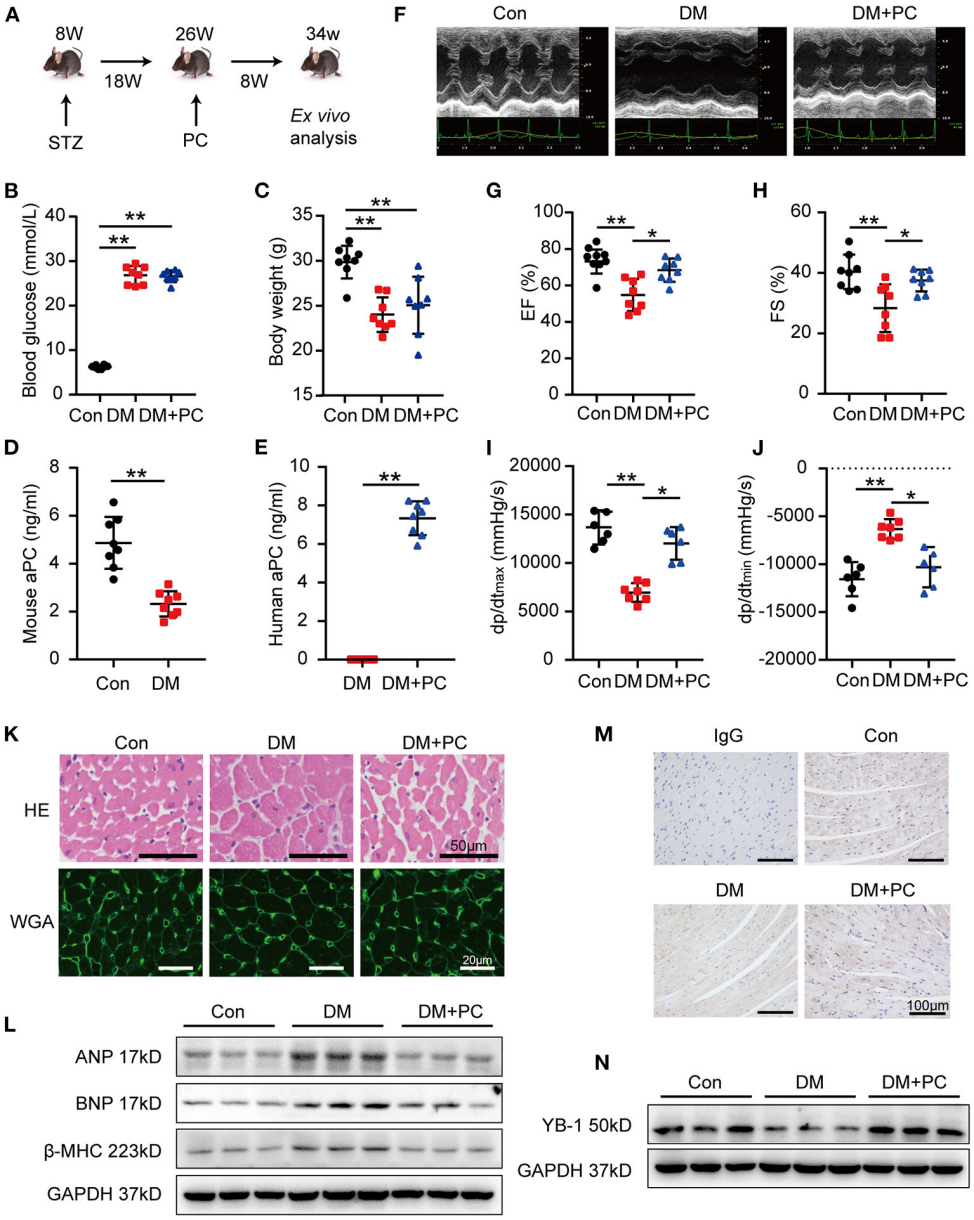
aPC protects DCM and sustains heart YB-1 expression. **(A)** Schematic diagram of animal experiment procedure. DCM was induced by a 5-day continuous intraperitoneal injection of STZ at the dose of 60 mg/kg. Treatment with exogenous PC 1 mg/kg/day intraperitonially was initiated at 18 weeks post-STZ intraperitoneal injection and lasted for 8 weeks. **(B)** Fasting blood glucose detection. Blood glucose was detected *via* tail vein. **(C)** Body weight was measured at 34 weeks. **(D)** Plasma endogenous mouse activated protein C level of controls and diabetic mice. **(E)** Plasma human activated protein C level of diabetic mice with or without exogenous human protein C intervention. **(F)** Representative echocardiographic images and **(G,H)** scatter plot summarized results of left ventricle ejection fraction (EF) and fraction shorting (FS). **(B–E,G,H)** *n* = 8 for all the groups. **(I,J)** Hemodynamic analysis measured by Millar cardiac catheter system of mice. (**I,J**, *n* = 6, Con; *n* = 7, DM; *n* = 6, DM + PC). **(K)** Exemplary images of hematoxylin-eosin staining (top, scale bar: 50 μm), wheat germ agglutinin staining (bottom, scale bar: 20 μm) of mouse hearts section. **(L)** Representative immunoblots showing the protein abundance of ANP, BNP, β-MHC in heart tissues from diabetic mice treated with PC or not, *vs*. controls. Total proteins were normalized to GAPDH. **(M)** Representative YB-1 immunohistochemical (IHC) staining of mice hearts tissue. Non-specific IgG served as negative control. scale bar: 100 μm. **(N)** Typical western blots of YB-1. GAPDH served as loading control. Data were presented as mean ± SD. Con, control; DM, diabetes mellitus; DM + PC, diabetes mellitus with PC treatment. *P < 0.05, **P < 0.01. *t-*test **(D,E)** or one-way ANOVA, Bonferroni comparison test **(B,C,G–J)**.

To ascertain the effect of protein C in DCM, we employed a well-established STZ induced type 1 DM mouse model, following with a daily exogenous PC treatment for 8 weeks starting from the 18th week after STZ administration (Figure 1A). The blood glucose levels of diabetic mice with or without PC treatment remained significantly higher than the non-diabetic, vehicle treated controls (Figure 1B). Meanwhile, the plasma insulin in STZ induced diabetic mice was significant lower and PC treatment made no improvement on it (Supplementary Figure S1A). Moreover, the triglyceride and total cholesterol levels in diabetic mice were elevated and PC treatment failed to regulate them (Supplementary Figures S1B,C). Taken together, these data demonstrated that the protective effect of PC was not linked to blood glucose, insulin or lipid regulation. The total body weight of diabetic mice was much lower and the ratio of heart to body weight was higher when compared with controls, while PC treated mice showed a moderate increase in body weight which was not statistically significant (Figure 1C; Supplementary Figure S1D).

To investigate whether exogenous PC supplement was activated *in vivo*, endogenous mouse aPC and exogenous human aPC were measured *via* commercially available ELISA kits (Figures 1D,E). Endogenous aPC levels in diabetic mice were significantly lower than the non-diabetic control mice, which was in agreement with the previous report (7). Exogenous human PC administration resulted in elevated human aPC levels in diabetic mice which conferred cytoprotective effects in DCM.

Echocardiography and hemodynamic analysis in diabetic mice showed significantly reduced left ventricular ejection fraction (EF), fractional shortening (FS), maximal rates of rise of ventricular pressure (dp/dt max) and maximal rates of decline of ventricular pressure (dp/dt min) (Figures 1F–J). Remarkably, exogenous administration of PC rescued cardiac dysfunction. Hematoxylin-eosin (HE) and wheat germ agglutinin conjugate (WGA) staining revealed an increased cardiomyocyte transverse cross-sectional area and a reduced number of cardiomyocytes in diabetic mice, while PC treatment significantly reversed those changes (Figure 1K; Supplementary Figures S1E,F). The expression level of biomarkers for cardiac function, atrial natriuretic peptide (ANP), brain natriuretic peptide (BNP) and myosin heavy chain beta-subunit (β-MHC) in mouse heart tissues detected by western blot correlated with the above findings (Figure 1L; Supplementary Figure S1G). Taken together, exogenous PC supplement even after the disease onset preserved the cardiac function and ameliorated myocardial injury in diabetic mice.

In a mouse model of renal ischemia-reperfusion injury, we have shown that aPC can suppress the ubiquitination of YB-1 by maintaining the deubiquitinase OTUB1 expression (14). To investigate the underlying mechanism through which exogenous PC exerts its protective effect in DCM, we have determined the expression levels of YB-1 in mouse heart tissues. When compared to non-diabetic control mice, YB-1 protein levels were significantly reduced in diabetic mouse hearts. Immunohistochemical staining showed a cardiomyocyte specific reduction of YB-1 in diabetic mouse hearts when compared to non-diabetic controls. Remarkably, exogenous PC treatment maintained YB-1's expression and distribution (Figures 1M,N; Supplementary Figures S1H,I).

Given that PC supplement administration preserved YB-1 levels in diabetic hearts presumably through increased aPC generation, we focused on the mechanisms of aPC dependent YB-1 regulation *in vitro*. We found that high glucose (HG, 25 mmol/L) treatment decreased YB-1 protein levels in a time dependent manner (Supplementary Figure S2A). Whereas, pre-treatment with aPC preserved YB-1 expression in a dose dependent manner, with an optimal concentration at 20 nM (Supplementary Figure S2B). Surprisingly, aPC even at 0.2 nM was still significantly effective (Supplementary Figure S2B). Pre-treatment with aPC, but not PC preserved the YB-1 expression in H9c2 cells indicating that the protective effect of exogenous PC on DCM was primarily mediated *via* aPC generation *in vivo* (Supplementary Figure S2C).

### Protective Effect of aPC in Diabetic Cardiomyopathy Requires YB-1

**Figure 2:**
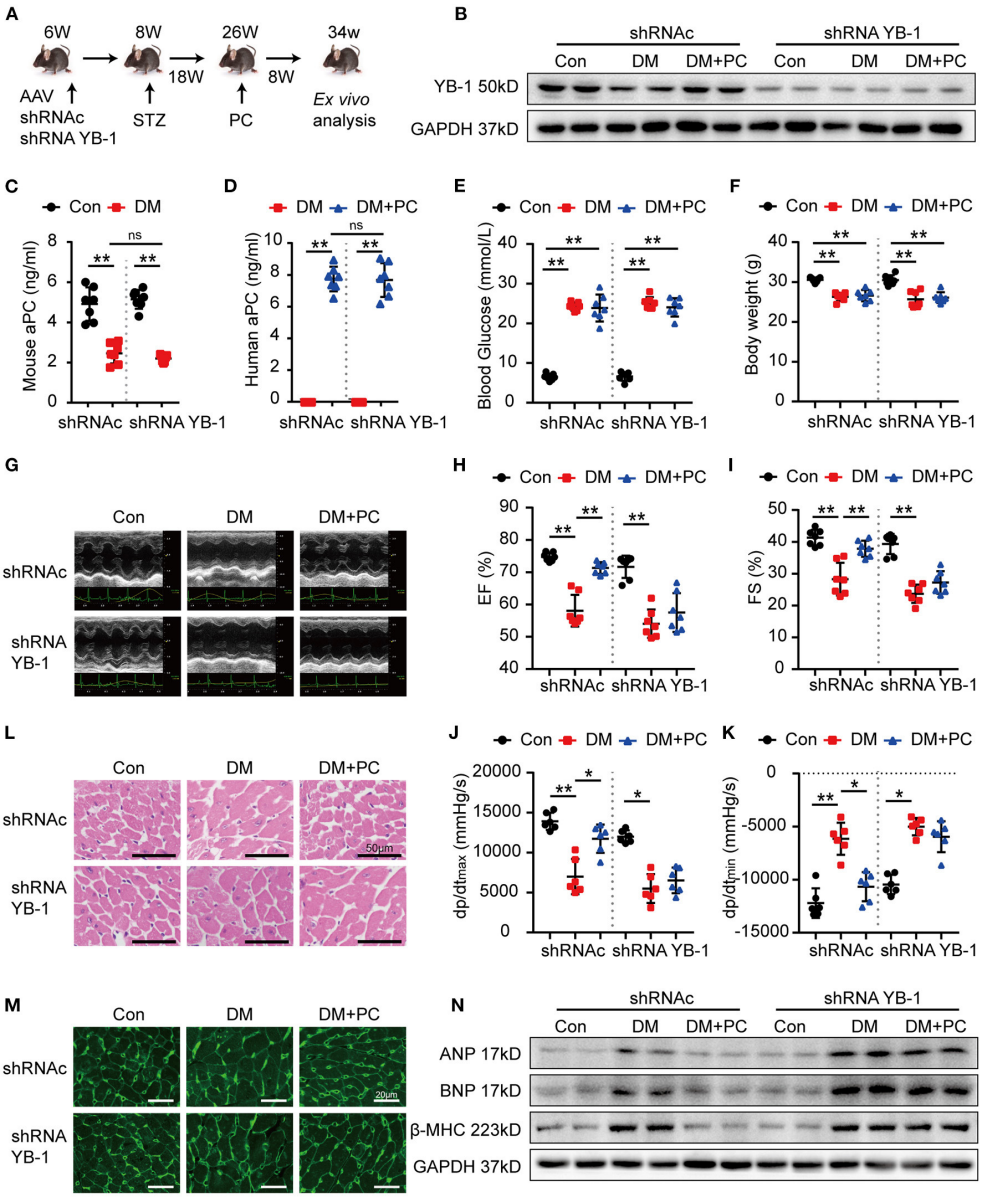
Protection of PC in DCM depends on YB-1. **(A)** Schematic diagram of animal experiment. C57/BL6J mice were first administrated with YB-1 shRNA AAV9 or scrambled shRNA AAV9 *via* tail vein at the age of 6 weeks, following with STZ injection and PC intervention at indicated time points. **(B)** Representative YB-1 immunoblots of mice heart tissue lysates verifying the knock down effect of YB-1 shRNA. **(C)** Plasma endogenous mouse aPC level and **(D)** exogenous human PC activation in indicated experimental groups were detected by commercially available ELISA kits. **(E)** Random blood glucose was detected among the experimental groups. **(F)** Body weight was measured at the age of 34 weeks. **(G)** Echocardiographic images of left ventricle and **(H,I)** scatter plots summarized ejection fraction and fraction shorting results. **(J,K)** Scatter plots summarized the hemodynamic parameters, which were measured by Millar cardiac catheter system. **(L)** Exemplary images of H&E-stained heart section. Scale bar: 50 μm. **(M)** Exemplary images of WGA-stained heart section. Scale bar: 20 μm. **(N)** Western blots showed the expression levels of ANP, BNP, β-MHC in heart tissue from different animal groups. Data were presented as mean ± SD, **(C–F,H,I)** *n* = 7 for all groups; **(J,K)** *n* = 6 for all groups. Con, control; DM, diabetes mellitus; DM + PC, diabetes mellitus with PC treatment; shRNAc, scrambled non-specific shRNA; shRNA YB-1, YB-1 specific shRNA; EF, ejection fraction; FS, fraction shorting. *P < 0.05, **P < 0.01. Student's *t*-test or two-way ANOVA, Bonferroni comparison test. ns, no significance.

To confirm the functional relevance of aPC-YB-1 axis for the observed cytoprotective effects in H9c2 cells, we generated YB-1 knock down H9c2 cells using YB-1 shRNA lentiviral particles (Supplementary Figures S3A,B). Although aPC pre-conditioning efficiently reduced the cardiac injury biomarkers expression in control H9c2 cells infected with lentivirus containing a scrambled shRNA, aPC failed to do so in YB-1 knock down H9c2 cells (Supplementary Figures S3C–F). To further investigate the role of YB-1 in aPC's protective effect *in vivo*, we used recombinant associated adenoviral vector rAAV (type 9) -YB-1-shRNA-GFP (Figure 2A). Intravenous administration of rAAV9-YB-1-shRNA-GFP in C57BL/6J mice *via* tail vein successfully resulted in a more than 60% reduction of YB-1 protein expression in the mouse hearts, as evidenced by western blot analysis (Figure 2B; Supplementary Figure S4A). In STZ-induced diabetic mice, YB-1 knock down *in vivo* has no effect on endogenous mouse aPC levels in control or diabetic mice (Figure 2C). Likewise, YB-1 knockdown in mice has no impact on *in vivo* aPC generation (Figure 2D), as well as blood glucose, plasma insulin, triglyceride, total cholesterol, body weight and ratio of heart weight to body weight (Figures 2E,F; Supplementary Figures S4B–E). Subsequently, echocardiograph and hemodynamic analysis were preformed (Figures 2G–K). When compared to diabetic mice infected with scrambled shRNA rAAV9, YB-1 knock down diabetic mice showed a tendency for lower cardiac function, which is not statistically significant. In agreement with the cytoprotective effects of aPC-YB-1 axis in H9c2 cells, exogenous PC intervention improved cardiac function of control diabetic mice, but it failed to do so in YB-1 knock down diabetic mice. HE and WGA staining showed that the downregulated YB-1 expression abolished the protective effect of PC on maintaining cardiomyocyte cell morphology (Figures 2L,M; Supplementary Figures S4F,G). Likewise, the expression level of ANP, BNP, β-MHC demonstrated that the protective effect of aPC dismissed in YB-1 knock down diabetic mice (Figure 2N; Supplementary Figures S4H–J). Thus, we can conclude that the protective effect of aPC in DCM is YB-1 dependent.

### YB-1 Protects Against Diabetic Cardiomyopathy *via* Transcriptional Suppression of MEF2B

**Figure 3:**
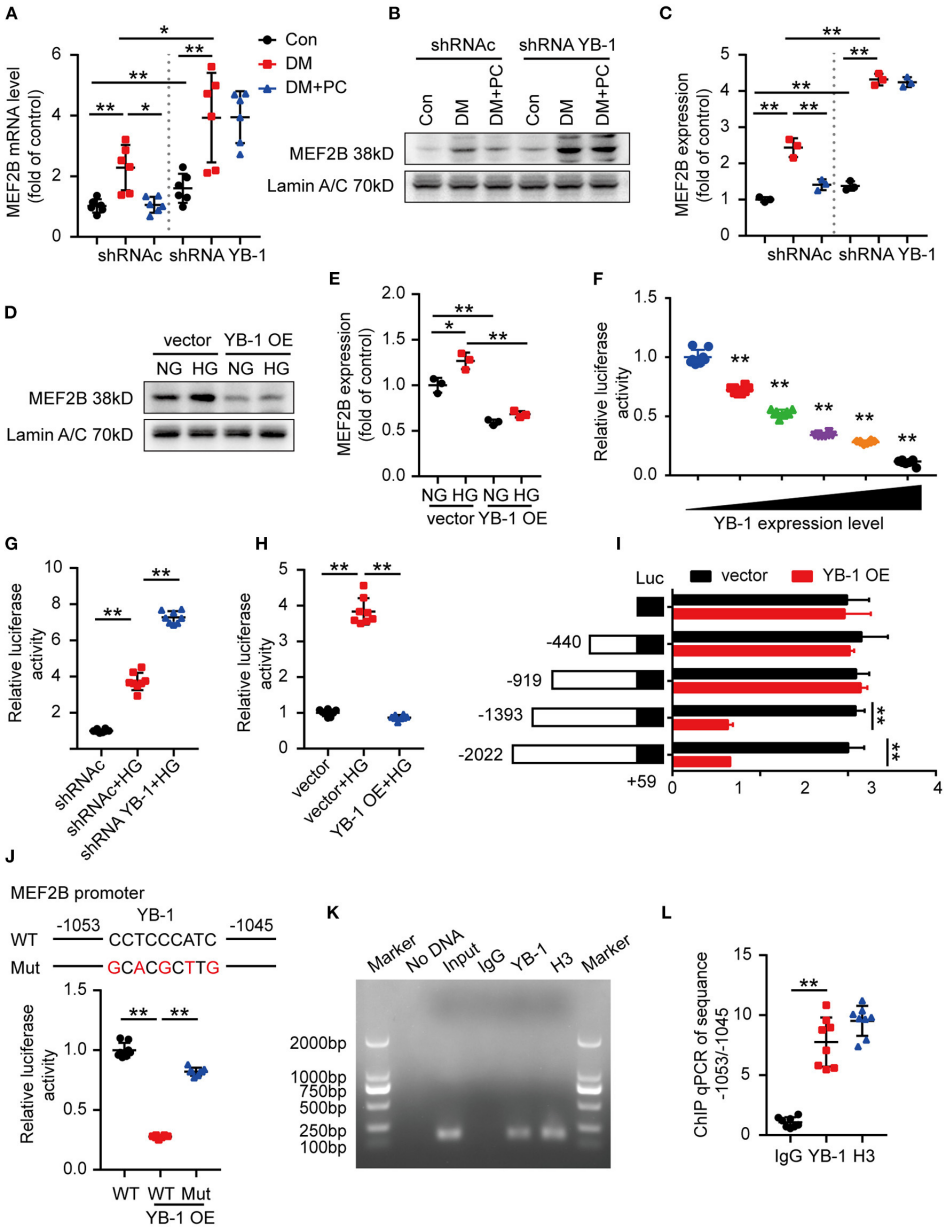
YB-1 may regulate cardiac function *via* MEF2B. **(A)** MEF2B mRNA level in mouse heart tissues detected by real-time qPCR. *n* = 6 for each group. **(B,C)** Representative western blots and scatter blots showing MEF2B nuclear protein level in mouse heart tissues from indicated experimental groups. **(D,E)** Representative immunoblotting and scatter blots demonstrating MEF2B nuclear protein level in H9c2 cells with or without YB-1 overexpression. **(B–E)** *n* = 3 for each group. **(F)** Luciferase activity of pGL3-MEF2B-promoter reporter plasmids stimulated by various YB-1 overexpression degree in H9c2 cells. **(G)** Luciferase activity of pGL3-MEF2B-promoter reporter in YB-1 knock down H9c2 cells or scrambled shRNA lentivirus infected H9c2 cells, stimulated with HG or not. **(H)** Luciferase activity of pGL3-MEF2B-promoter reporter plasmids in YB-1 overexpression H9c2 cells or controls, stimulated with HG or not. **(I)** Luciferase activity of pGL3 (−2,022/+59), pGL3 (−1,393/+59), pGL3 (−919/+59), pGL3 (−440/+59), and pGL3-basic reporter plasmids stimulated by YB-1 overexpression in H9c2 cells. **(J)** Schematic illustration of mutant of the binding sites of YB-1 on MEF2B promoter in the predicted region of −1,053 to −1,045 (upper part). Luciferase activity was measured in H9c2 cells with mutant or wild type vector stimulated by YB-1 overexpression (lower part). **(K,L)** ChIP (chromatin immunoprecipitation) result was represented by agarose gel electrophoresis and real time qPCR which revealed the interaction of YB-1 and the promoter region of MEF2B. **(F–L)** *n* = 8 for each group. Data were represented as fold of control, mean ± SD. Con, control; DM, diabetes mellitus; DM + PC, diabetes mellitus with PC treatment; shRNAc, scrambled non-specific shRNA; shRNA YB-1, YB-1 specific shRNA; NG, 5.5 mmol/L D(+) glucose; HG, 25 mmol/L D (+) glucose; OE, overexpression; WT, wild type; Mut, Mutant. *P < 0.05, **P < 0.01. Student's *t*-test or two-way ANOVA, Bonferroni comparison test.

A few transcription factors, such as MEF2A, MEF2B, MEF2D, STAT3, GATA4, SOX9, SOCS3, HSF1, and YAP have been recently shown to play important roles in cardiomyopathies (20–27). Therefore, we speculate that YB-1 may execute its protective effect in DCM by regulating some of the above-mentioned key transcription factors. Subsequently, we determined the gene expression of the key transcription factors in the mouse hearts. Intriguingly, among those candidate genes, MEF2B was found to be the mostly regulated one in the heart tissues of YB-1 knock down mice (Figure 3A; Supplementary Figure S5). Consistent with the mRNA level, MEF2B nuclear protein level was also markedly increased in heart lysates of the YB-1 knock down mice (Figures 3B,C). These data suggest that YB-1 expression may directly or indirectly restrains MEF2B levels. Given the crucial role of MEF2B in regulating cardiac function (28), we next investigated the potential role of YB-1 in regulation of MEF2B expression using YB-1 overexpressing H9c2 cells. HG treatment of control H9c2 cells upregulated nuclear level of MEF2B protein, whereas YB-1 overexpression suppressed MEF2B expression (Figures 3D,E).

The multifunctional YB-1 can execute its effects mainly *via* its transcription regulation (29). To determine whether YB-1 directly binds to MEF2B promoter, we designed pGL3-MEF2B-promoter reporter plasmid to evaluate the influence of YB-1 on MEF2B transcriptional regulation. Dual-luciferase assays showed an inverse correlation between YB-1 expression and luciferase activity of pGL3-MEF2B-promoter reporter plasmid (Figure 3F). On the other hand, HG stimulation or YB-1 knock down significantly elevated its luciferase activity (Figure 3G), while YB-1 overexpression suppressed MEF2B (Figure 3H).

To identify the specific binding sites through which YB-1 and MEF2B interacts, we generated four deletion mutants of the MEF2B promoter region using pGL3 luciferase reporter vector, pGL3 (−2,022/+59), pGL3 (−1,393/+59), pGL3 (−919/+59), pGL3 (−440/+59). YB-1 overexpression decreased the luciferase activity of the −2,022/+59 and −1,393/+59 deletion mutants, while made no difference to −919/+59 and −440/+59 mutants, which demonstrated that the binding site lies in the region of −1,391/−919 (Figure 3I). JASPAR database (http://jaspar.genereg.net/) was used to predict the binding motif of YB-1 and MEF2B promoter and identified −1,053/−1,045 as the potential binding site. Thus, a mutant pGL3-MEF2B-promoter reporter plasmid was cloned by replacing the −1,053/−1,045 region (CCTCCCATC) with a partially complementary one (GCACGCTTG). YB-1 overexpression made no difference to the mutant pGL3-MEF2B-promoter reporter luciferase activity, which confirmed −1,053/−1,045 region as binding site of YB-1 within the MEF2B promoter (Figure 3J). The regulatory effect of −1,053/−1,045 region was further determined by chromatin immunoprecipitation real time qPCR (Figures 3K,L). These data demonstrate that the cardioprotective effect of YB-1 is dependent on the regulation of MEF2B transcription.

### Activated PC Maintains YB-1 Expression *via* OTUB1

**Figure 4:**
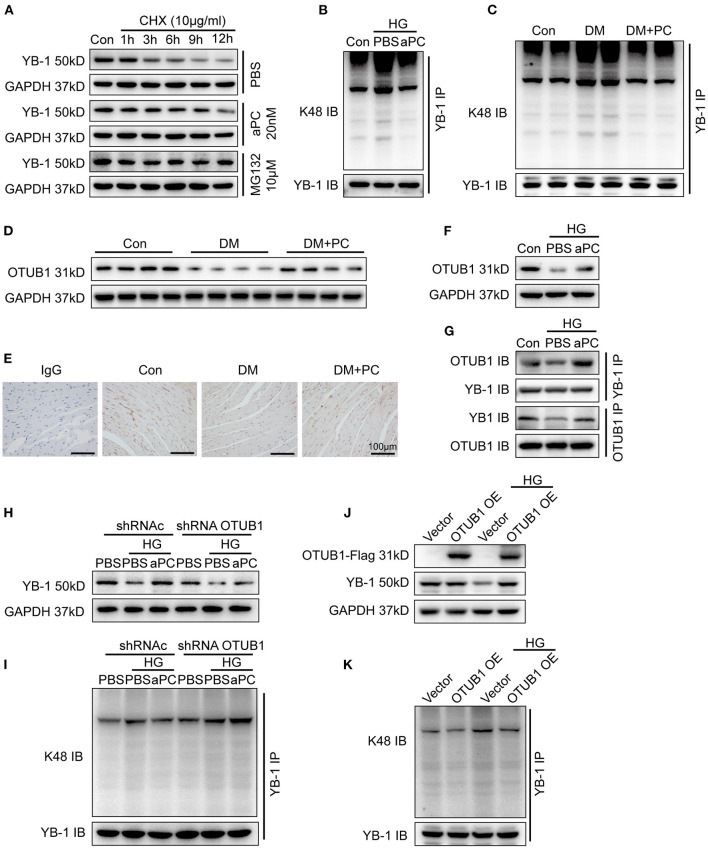
aPC can maintain the YB-1 expression *via* OTUB1 dependent K48 deubiquitination. **(A)** After protein synthesis inhibition by CHX (10 μg/ml) treatment, YB-1 protein stability was enhanced by pre-treatment of proteasome inhibitor MG132 (10 mM) and aPC (20 nM) in H9c2 cells. **(B)** Co-IP showed that HG can promote the K48-linked ubiquitination of YB-1 in H9c2 cells infected with GFP-labeled YB-1 overexpression adenovirus, while aPC can reverse it. Co-IP was performed with 1 μg anti-GFP primary monoclonal antibody. **(C)** K48-linked ubiquitination of YB-1 in diabetic mice heart tissue with or without PC treatment. Co-IP was performed with 1 μg anti-YB-1 monoclonal antibody. **(D)** Representative OTUB1 immunoblots in mice heart tissue lysates with or without PC treatment. **(E)** Immunohistochemistry staining showing the expression level of OTUB1 in mice heart section. Scale bar: 100 μm. **(F)** Exemplary OTUB1 immunoblots in HG co-incubated H9c2 cells challenged with aPC or not. **(G)** H9c2 cells were infected with both GFP-labeled YB-1 overexpression adenovirus and Flag-labeled OTUB1 overexpression adenovirus. Co-IP was performed with corresponding antibodies to determine the interaction between YB-1 and OTUB1. **(H)** Representative immunoblots of YB-1 in H9c2 cells infected with control shRNA lentivirus or OTUB1 specific shRNA lentivirus. **(I)** Co-IP result showed K48-linked ubiquitination of YB-1 in OTUB1 knock down H9c2 cells. **(J)** Western blots of YB-1 in H9c2 cells infected with control adenovirus or OTUB1 overexpression adenovirus, stimulated with HG or not. H9c2 cells were infected with vector control adenovirus or Flag-labeled OTUB1 overexpression adenovirus. Antibody to Flag was used to detect the exogenous overexpressed protein. **(K)** Overexpressing OTUB1 could ameliorate the K48-linked ubiquitination of YB-1. Con, control; MA, mannitol; HG, 25 mmol/L D (+) glucose; aPC, activated protein C; PC, protein C; shRNAc, scrambled non-specific shRNA; shRNA YB-1, YB-1 specific shRNA; shRNA OTUB1, OTUB1 specific shRNA; CHX, cycloheximide; DM, diabetes mellitus; DM + PC, diabetes mellitus with PC treatment; OE, overexpression. The above blots were performed at least in triplicate.

In a mouse model of renal ischemia reperfusion injury, we have demonstrated that aPC modulates YB-1 expression by regulating its protein stability *via* deubiquitinase enzyme OUTB1 (14). Accordingly, we determined the regulatory effect of aPC on YB-1 protein stability in cardiomyocytes. Cycloheximide (CHX) was used to inhibit the protein synthesis in H9c2 cells and cell permeable reversible proteosome inhibitor MG132 intervention was used as positive control to evaluate YB-1 protein stability. The natural degradation of YB-1 protein was observed in PBS treated control group, which was preserved by proteasome inhibitor MG132 treatment. Akin to proteosome inhibitor MG132, aPC treatment but not vehicle (PBS) prevented YB-1 protein degradation (Figure 4A; Supplementary Figure S6A). These data suggest that aPC maintains YB-1 protein levels through preventing its proteasomal degradation. To ascertain this hypothesis, we determined YB-1 K48 linked polyubiquitination, a predominant mechanism of proteasomal degradation (30). When compared to mannitol control, K48 linked polyubiquitination was markedly increased by HG stimulation, whereas aPC treatment inhibited ubiquitination (Figure 4B). In agreement with this observation, we found a similar K48 linked polyubiquitination pattern of YB-1 in mouse heart tissue lysates with DCM (Figure 4C).

To further explore the underlying mechanism of YB-1 ubiquitination, protein level of deubiquitinating enzyme otubain-1 (OTUB1) in diabetic mouse heart tissue was determined. A significantly reduced expression of OTUB1 was shown in diabetic mice, whereas PC treatment maintained OTUB1 levels (Figures 4D,E; Supplementary Figures S6B,C). Congruently, aPC preserved OTUB1 levels in HG treated H9c2 cells (Figure 4F). Consistent with hyperglycemia-induced reduced expression of YB-1 in H9c2 cells and in diabetic mice, Co-IP studies showed an impaired interaction between YB-1 and OTUB1 in H9c2 cells incubated with HG, whereas aPC treatment significantly preserved YB-1/OTUB1 interaction (Figure 4G; Supplementary Figure S6D). Although aPC retained YB-1 protein level in H9c2 cells infected with scrambled shRNA under HG stimulation, it failed to do so in OTUB1 knock down cells (Figure 4H; Supplementary Figure S6E). Likewise, aPC failed to prevent K48 linked polyubiquitination of YB-1 in OTUB1 knock down H9c2 cells (Figure 4I). These data indicate that OTUB1 lies downstream of aPC signaling and is required for aPC-dependent YB-1 regulation. Accordingly, overexpression of OTUB1 in H9c2 cells was sufficient to prevent HG induced ubiquitination and thereby reduced expression of YB-1 levels (Figures 4J,K; Supplementary Figure S6F).

### Activated Protein C Maintains YB-1 and OTUB1 Expression Through PAR1 and EPCR

**Figure 5:**
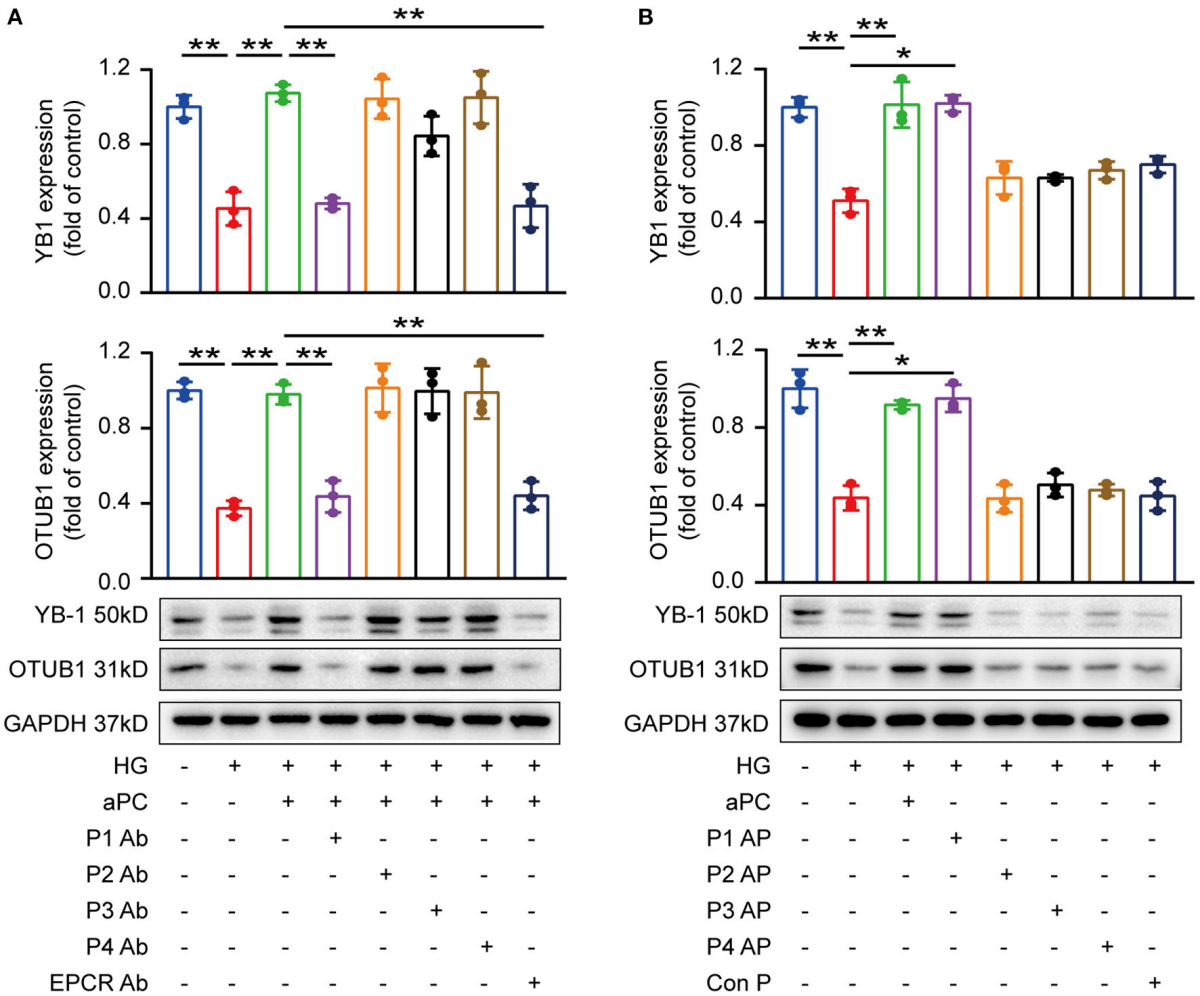
aPC maintains YB-1 and OTUB1 expression *via* PAR1 and EPCR. **(A)** H9c2 cells were pre-treated with specific blocking antibodies (20 μg/ml, 1 h) then aPC (20 nM, 30 min), and thereafter HG (25 mM, 6 h). Western blots showed that PAR1 and EPCR blocking antibodies abolished the protective effect of aPC on maintaining the expression of YB-1 and OTUB1. **(B)** H9c2 cells were pre-treated with PAR agonists or control peptide (10 μM, 1 h). PAR1 agonist could maintain YB-1 and OTUB1 expression. Data were represented as fold of control, mean ± SD. *n* = 3 for all groups. HG, 25 mmol/L D (+) glucose; aPC, activated protein C; P1-4, Protease activated receptor 1–4; EPCR, Endothelial protein C receptor; Ab, antibody; AP, agonist peptide. *P < 0.05, **P < 0.01. Student's *t-*test, Bonferroni comparison test. All the blots were performed at least in triplicate.

On endothelial cells, cytoprotective aPC signaling is mediated by endothelial protein C receptor (EPCR) dependent protease activated receptor-1 (PAR-1) activation (31, 32). To identify the receptors dependent mechanisms involved in cardiomyocytes, we determined the expression of PARs and EPCR in mice heart tissue and H9c2 cardiomyocytes by immunoblotting and immunofluorescence (Supplementary Figure S7). Blocking antibodies against PAR1 and EPCR efficiently diminished the effect of aPC on preserving YB-1 and OTUB1 expression, whereas no effect was observed in PAR2, PAR3, and PAR4 blocking antibodies (Figure 5A). In congruence with aPC/EPCR/PAR1 dependent regulation of YB-1, direct activation of PAR1 using PAR1 receptor agonist improved YB-1 and OTUB1 protein levels (Figure 5B). Thus, these results conclude that aPC signaling *via* EPCR/PAR1 regulates YB-1 and OTUB1 protein expression.

## Discussion

DCM has attracted more attention (33–35) as traditional anti-heart failure therapy and blood glucose control cannot completely reverse or alleviate DCM, making it a high priority unmet clinical need. In this study, we identified the protective effect of aPC on DCM. Protein C is an important natural anticoagulant, and its activated form, aPC has been reported to be protective in multiple pre-clinical models including diabetic nephropathy and a series of heart-related diseases (9, 36, 37). Our data showed that the administration of exogenous PC in mice effectively increased the systemic aPC levels and improved cardiac function and protected against DCM. Mechanistically, aPC signaling *via* PAR1 and EPCR induces the key deubiquitinating enzyme OTUB1 which preserves YB-1 expression. Maintenance of YB-1 levels restrained MEF2B transcription and protected from DCM. This newly identified cardioprotective mechanism not only provide novel mechanistic insights into the regulation of DCM but also demonstrate that targeting this pathway, *e.g*., with exogenous PC, ameliorates chronic DCM.

As a vitamin-K dependent plasma anticoagulant, aPC has both anti-inflammatory and cytoprotective properties (38), and recombinant aPC was once approved by the FDA for the treatment of severe sepsis, unfortunately was retracted from the market partially because of its severe bleeding complications (39, 40). Beyond its anti-sepsis effect, aPC and its variants have been shown to be beneficial in various pre-clinical cardiac disease models including myocardial ischemia-reperfusion injury (IRI) (41–43), myocardial fibrosis (44) and pressure overload-induced hypertrophy (45). The current study reveals for the first time the underlying molecular mechanism and protective effect of aPC in a chronic mouse model of DCM. The cytoprotective effect of aPC is predominantly mediated *via* G protein coupled receptors, PAR1 and PAR3. On endothelial cell surface, aPC bound to EPCR transmits biased, PAR1/β-arrestin-dependent cytoprotective signaling. On specialized renal epithelial cells (podocytes), aPC-bound to integrin β3 mediates its cytoprotective responses *via* PAR3. Engagement of cell-specific receptor complexes transmits diverse intracellular responses that involves signaling *via* distinct small G proteins, β-arrestins, sphingosine-1 phosphates, and disabled-1 (13, 46). Previously we have shown that aPC protects against renal IRI by maintaining OTUB1 and YB-1 expression in tubular cells *via* its classical receptor PAR1 and EPCR. While these studies have identified YB-1 as a novel intracellular target for aPC (14).

The underlying mechanism by which YB-1 performs its cytoprotective effects remains unclear (14). The multifunctional YB-1 is a highly conserved protein and can execute its effects mainly *via* its transcription regulation (29). Among the transcription factors, MEF2A, MEF2B, MEF2D, STAT3, GATA4, SOX9, SOCS3, HSF1, and YAP (20–27) which play important roles in cardiomyopathies, we found that MEF2B was a downstream target of YB-1 in cardiomyocytes. MEF2B belongs to the MEF2 (Myocyte Enhancer Factor 2) family of transcription factors, which includes three additional members, MEF2A, MEF2C and MEF2D, is thought to play an important role in differentiation of cardiomyocytes (47). MEF2B has essential roles in maintaining cell reproduction, cell growth and plays an important role in multiple pathways relevant to DNA replication and repair, cell cycle and apoptosis (48). Recent study has reported that MEF2B promotes mitochondrial dysfunction, thus accelerating cardiac hypertrophy and heart failure (27). The current results show that YB-1 regulates MEF2B expression *via* binding to its −1,053 to −1,045 promoter region which confers cytoprotective effect in DCM.

In agreement with our previous report in renal tubular cells (14), we could show that on the one hand high glucose deceased the protein expression of deubiquitinase OTUB1 and its interaction with YB-1, while aPC could maintain its expression and increase the interaction with YB-1. On the other hand, OTUB1 overexpression or knock down had significant effects on YB-1 ubiquitination level on K48 linked polyubiquitin chain and its protein level in cardiomyocytes. Thus, aPC-dependent YB-1 regulation requires OTUB1. However, how aPC regulates OTUB1 needs further elucidation. Recent studies have shown that the CXCR7/ERK1/2 signaling pathway can increase OTUB1 expression and improve the stability of endoplasmic reticulum (49). ERK can directly bind to the promoter region of OTUB1 to regulate its expression in colorectal cancer (50). MicroRNA-542-3p can increase OTUB1 mRNA and protein levels to inhibit the proliferation, migration, and invasion of colorectal cancer cells. In addition, studies have found that OTUB1 can regulate its interaction with RhoA, increasing its stability and activity, and thus enhance the susceptibility of host cells to *Yersinia plague* (51). Furthermore, OTUB1 phosphorylation can maintain its stability and promote its interaction with RhoA protein to maintain RhoA protein expression (51). Therefore, we speculate that aPC may regulate OTUB1 expression *via* the following mechanisms: (1) ERK1/2 signaling pathway; (2) MicroRNA expression, such as MicroRNA-542-3p; (3) OTUB1 phosphorylation; (4) Interaction with YB-1. Additional investigations are required to uncover the specific mechanism by which aPC regulates the expression and function of OTUB1.

Independent of its anticoagulant property, aPC has been shown to confer protective effects in various acute and chronic pre-clinical models. The current study identifies an unrecognized function of aPC in regulation of proteasomal degradation of YB-1 and thereby restrain MEF2B promoter activity and protect against DCM. Our study demonstrates that targeting OTUB1/YB-1/MEF2B pathway ameliorates DCM.

## Data Availability Statement

The original contributions presented in the study are included in the article/Supplementary Material, further inquiries can be directed to the corresponding author/s.

## Ethics Statement

The animal study was reviewed and approved by the Institutional Animal Research Committee of Tongji Medical College.

## Author Contributions

HW and HZ conceptually designed the work. TM and XH interpreted the data and critically reviewed the manuscript. XZ and TW conducted the main work and prepared the manuscript. YX and MW assisted *in vivo* work and analyzed the data. WZ and LD assisted animal sample collection and analysis. JL conducted the virus administration. XN conducted the hemodynamic measurements. All authors contributed to the article and approved the submitted version.

## Funding

This work was supported by the National Natural Science Foundation of China (No. 81600301 to HW; Nos. 81873523, 82070490 to HZ; No. 81800411 to XH; No. 51707076 to JL) and the Fundamental Research Funds for the Central Universities (HUST: 2020JYCXJJ018; 2021yjsCXCY096 to XZ).

## Conflict of Interest

The authors declare that the research was conducted in the absence of any commercial or financial relationships that could be construed as a potential conflict of interest.

## Publisher's Note

All claims expressed in this article are solely those of the authors and do not necessarily represent those of their affiliated organizations, or those of the publisher, the editors and the reviewers. Any product that may be evaluated in this article, or claim that may be made by its manufacturer, is not guaranteed or endorsed by the publisher.
