# Supplementary material for: Activated Protein C Ameliorates Diabetic Cardiomyopathy via Modulating OTUB1/YB-1/MEF2B Axis
Source: Front Cardiovasc Med. 2021 Oct 29;8:758158. doi: 10.3389/fcvm.2021.758158 (PMC8585767; doi:10.3389/fcvm.2021.758158)
Supplement: Supplementary file 1 [file Data_Sheet_1.docx]

**SUPPLEMENTAL MATERIALS**

**1. Supplementary materials and methods**

**1.1 Materials**

The following antibodies were used in the current study: Rabbit polyclonal antibodies to ANP, BNP, β-MHC were from ABclonal Technology; rabbit polyclonal antibody to YB-1 was from CUSABIO; rabbit monoclonal antibody to YB-1 was from Abcam; rabbit monoclonal antibody to H3, OTUB1, mouse monoclonal antibodies to IgG, flag, ubiquitin, rabbit polyclonal antibody to K48-linkage specific polyubiquitin were from Cell Signaling Technology; rabbit polyclonal antibodies to PAR1, PAR2, PAR3, PAR4, MEF2B were from Santa Cruz; rabbit polyclonal antibody to EPCR was from BOSTER Biological Technology; mouse monoclonal antibody to EPCR was from Sigma-Aldrich; rabbit polyclonal antibody to GAPDH, rabbit anti-mouse IgG-HRP conjugated secondary antibody and mouse anti-rabbit IgG-HRP conjugated secondary antibody were from Servicebio.

The following reagents were used in the current study: streptozotocin, MG132, D-(+)-Glucose solution, D-Mannitol were from Sigma-Aldrich; fetal bovine serum, low glucose DMEM, DMEM were from Gibco; protein A/G agarose beads was from Santa Cruz; protease inhibitor cocktail and phosphatase inhibitor cocktail were from MedChemExpress; RNAiso Plus was from Takara; antibiotic solution, enhanced chemiluminescence reagent, BCA reagent were from BORTER Biological Technology; PVDF membrane was from Millipore GmbH; transfection reagent Turbofect was from Thermo Fisher Scientific; OTUB1 ORF overexpression plasmid, shRNA vectors for YB-1 and OTUB1 were from Thermo scientific and OriGene, respectively; Restriction Enzymes, T4 DNA ligase, cycloheximide were from New England Biolabs; mouse PARs agonists and control peptides were from Bachem; DAB substrate Kit for peroxidase was from Vector Laboratories; ABScript II reverse transcription premix and Genious 2X SYBR Green Fast qPCR Mix were from ABclonal Technology; lepirudin was from celgene; Dual luciferase assay was from Promega; ChIP assay kit, Quick Mutation Site-Directed Mutagenesis Kit were from Beyotime; mouse or human activated protein C ELISA Kits were from CUSABIO; insulin, triglyceride and total cholesterol Assay Kits were from Nanjing Jiancheng Bioengineering Institute, Nanjing, China.

**1.2 Animal model for diabetic cardiomyopathy**

Type 1 diabetes mellitus model was induced by a 5-day continuous intraperitoneal injection of streptozotocin (STZ) at the dose of 60 mg/kg to 8-week C57BL6/J mice and mice received equal volume of vehicle severed as control[1]. STZ (Sigma-Aldrich, 500mg) was dissolved in 33ml sterilized pH 4.5, 0.05M citrate sodium solution. Blood glucose was first detected in 2 weeks post STZ injection *via* glucose sticks (Onetouch Ultra). Mice with blood glucose higher than 16.7 mmol/L were considered as diabetes mellitus. The blood glucose and body weight were monitored weekly. Mice with blood glucose higher than 27.7 mmol/L received a subcutaneous injection of 0.5U insulin Lantus twice a week to avoid death from excessive hyperglycemia. Cardiac function was determined by echocardiography and hemodynamic analysis, diabetic mice with impaired cardiac function were defined as diabetic cardiomyopathy (DCM)[2]. To evaluate the effect of exogenous PC supplement on diabetic cardiomyopathy, mice received 1mg/kg PC intraperitoneal injection daily starting from 18w to 26w post STZ injection.

**1.3 Echocardiography and** **hemodynamic measurements**

To determine the cardiac function of mice, echocardiography and hemodynamic measurements were employed[2,3]. Echocardiography was performed by ultrasound professionals with a VisualSonics vevo 770 imaging system (VisualSonics, Toronto, Canada). In brief, all mice were anesthetized with 0.5% isoflurane and placed on the test bench in the supine position. Hairs on the chest were removed. Left ventricular ejection fraction and fractional shortening data were obtained from M-mode with a stable heart rate from 500bpm to 600bpm.

To evaluate the cardiac function more accurately, a catheter manometer (Millar Instruments) was inserted into the mice left ventricle through its right carotid artery after it was anesthetized by 1% pentobarbitone sodium. Pentobarbitone sodium was intraperitoneally injected at the dose of 80mg/kg per mice. Hemodynamic parameters were recorded consecutively. Maximal rates of rise of ventricular pressure (dP/dt _max_), and maximal rates of decline of ventricular pressure (dP/dt _min_) were calculated with the PVAN software.

**1.4 Histology, immunohistochemistry and immunofluorescence**

After the Millar catheter measurement, an excessive pentobarbitone sodium solution intraperitoneal injection was performed. Sacrificed mouse blood sample was collected into a centrifuge tube with 0.38% sodium citrate and 50 mM benzamidine HCl. Sacrificed mouse heart was perfused with ice-cold PBS to remove the residual blood. Mouse hearts were fixed for 24 h-48 h with 4% paraformaldehyde (ServiceBio), embedded in paraffin and sectioned into 4μm slices. To evaluate the degree of myocyte hypertrophy, hematoxylin-eosin and wheat germ agglutinin staining were performed. Heart sections were first deparaffinized with xylol, dehydrated with graded ethanol and washed by PBS several times. For H&E staining, heart section was incubated with corresponding dye solution for 10 min at room temperature. For WGA staining, heart section was incubated with 200μg /ml WGA dye solution for 1 h at 37℃.

As for immunohistochemical staining, antigens were retrieved by heating the heart section with 0.5M citrate (pH 6.0) after its deparaffination and dehydration. The heart sections were incubated with peroxidase substrate (3%) for 30 minutes, blocked with goat serum for 1 h at room temperature. Primary antibody to YB-1 or primary antibody to OTUB1 was used to detect the specific antigens. After incubating with primary antibody at 4℃ overnight, corresponding secondary antibodies conjugated with HRP were employed. Immunolabels were demonstrated by 3, 3′-diaminobenzidine (DAB) and nuclei counterstained with hematoxylin. Tissue sections were visualized under microscope (Olympus) at 400× magnification.

As for immunofluorescence, H9c2 cells were fixed with 4% paraformaldehyde for 15 min, incubated with enhanced immunostaining permeabilization buffer (P0097, Beyotime Biotechnology) for 15 min and blocked with 10% goat serum (E510009, Sangon Biotech) for 1 h. Then, H9c2 cells were incubated with mouse monoclonal antibody to EPCR (WH0010544M3, Sigma- Aldrich) at the ratio of 1: 100 at 4℃ overnight, followed by FITC-labeled Goat Anti-Mouse IgG (A0568, Beyotime Biotechnology) and DAPI (C1002, Beyotime Biotechnology) incubation for 30 min. Fluorescence images were obtained under microscope (Olympus) at 400× magnification and analyzed with Image-Pro Plus 6.0 software.

**1.5 ELISA**

Plasma aPC and insulin levels were determined by mouse or human activated protein C ELISA Kit (CSB-E09909h, CSB-E09914m, Cusabio, Wuhan, China) and insulin ELISA Kit (H203-1-2, Nanjing Jiancheng Bioengineering Institute, Nanjing, China), according to the operating manuals[4,5]. In details, a capture antibody specific for aPC or insulin was coated onto the wells of microplates in advance. Prepared standard of graded concentration was used to make a standard curve. Plasma samples, control specimens, and standards were gently pipetted into these wells in equal volume and incubated for 30 min at 37 ℃. After washing for 5 times, a labeled enzyme reagent was added into the wells for a second 30 min incubation. Chromogenic substrate was added and bound to the detection antibody, following by a third incubation for 15 min in the dark at room temperature. Stop solution was used and the solution in wells changed from blue to yellow. The absorbance of this colored product at 450nm was detected directly. The concentration of aPC or insulin in different samples was proportional to its absorbance according to the standard curve. Each sample was measured twice to get a stable result.

According to the manufacturers' instructions, the test kits (A110-1-1 for triglyceride assays and A111-1-1 for cholesterol assays, Nanjing Jiancheng Bioengineering Institute) were used to determine the lipids extracted from serum.

**1.6 Immunoprecipitation and immunoblotting**

To get mice heart tissue lysates, mice hearts were snap frozen in liquid nitrogen, ground into powder and collected. RIPA buffer was prepared in advance mixed with protease inhibitor cocktails and was added into the tissue powder. After a 30 min incubation on ice, mixture was centrifugated at the speed of 12,000g for 25 min. Supernate was collected to a new centrifuge tube for further experiment. To get the cultivated cell protein, cells were first washed with cold PBS for 3 times to remove the culture medium completely. Proteins were then extracted using RIPA buffer with a 30 min incubation and a 25 min centrifugation at the speed of 12,000g. The concentration of protein was determined by BCA reagent kit[6].

For immunoprecipitation[7], part of protein lysates (around 10%) was used as input control. The remaining protein lysates were precleared with 20μl protein A/G agarose to avoid unspecific binding. Cleared protein lysates were then mixed with 1 μg antibody to YB-1/GFP, OTUB1/Flag or irrelevant IgG antibody and incubated for 1h at 4 ℃. 30μl protein A/G agarose beads were used to precipitate the antigen-antibody complex at 4 ℃ overnight. Complex was washed with cold PBS or RIPA for 3 times the next day and stored at -80℃ or used for immunoblotting immediately.

After being boiled for 5 - 10 min, equal amounts of protein lysates or precipitates were separated by SDS/PAGE electrophoresis and transferred to polyvinylidene difluoride (PVDF) membranes. After blocking with 5% BSA at room temperature for 1 h, membranes were incubated with specific primary antibodies at 4℃ overnight and were incubated with appropriate secondary HRP-conjugated IgG antibodies at room temperature for 1 h the next day. The immunoblotting bands were visualized with an enhanced chemiluminescence reagent kit and analyzed by image J Acquisition.

**1.7 Cell culture and treatment**

The H9c2 cell line and 293T cell line were purchased from the American Type Culture Collection (ATCC) and cultured in low glucose DMEM supplemented with 10% fetal bovine serum (FBS) and 1% penicillin/streptomycin solution[6]. Cells were routinely cultured at 37℃ with 5% CO_2_ and were starved in serum-free low glucose DMEM for 6 h before any intervention. To mimic a hyperglycemia environment, high glucose culture medium at the final concentration of 25 mmol/L glucose was used, the same osmotic pressure mannitol severed as control[8]. Different concentration of aPC was used to treat the H9c2 cells and 293T cells for certain periods of time before the high glucose intervention to determine the most appropriate dose and time points. Meanwhile, lepirudin (1μg/ml, final concentration) was used to avoid possible side effect of thrombin contamination during aPC manufacture[9]. At the optimal dose and time points post aPC and HG treatment, protein lysates and total RNA were extracted for immunoblotting, real-time PCR and ChIP. To explore the effect of aPC on YB-1 degradation, CHX (10μg/ml) was used to inhibit protein synthesis and MG132 (10μM) was used to inhibit protein degradation[10]. PARs and EPCR blocking antibodies (10μg/ml) and agonist (10μM) were used to pretreat H9c2 cells 30 min before aPC treatment and followed by high glucose stimulation[11]. PARs agonist and control peptides sequences were listed in Supplementary Table S1.

**1.8** **Generation of knockdown cell lines**

Stable knockdown cell lines were generated as previously described[12]. The three plasmids co-transfect system was employed to produce different lentivirus, including shRNA-YB1 or shRNA-OTUB1. Briefly, YB-1, OTUB1, or Scrambled shRNA plasmids (pLKO.1-puro vector, knock down effect verified) along with PMD.2g and psPAX2 plasmids transfected into HEK293 cells. Culture medium was refreshed 6 h post transfection and was collected 48 h post transfection. After a centrifugation at the speed of 12,000g, supernatant containing lentivirus was collected and used to infect H9c2 cells. Culture medium containing 1mg/ml puromycin was employed to consistently select the cell lines infected. After selecting for 3 generations, cells were seeded into 96 well plates with individual clones. Cells were reproduced for several generations. Western blotting and real-time PCR were applied to single out the clones with low expression target genes.

**1.9 Quantitative real time PCR**

Total RNA of mouse heart tissue was extracted with Trizol reagent[13]. RNA was dissolved in nuclease-free water. The concentration of RNA was determined by the absorbance of A260 and A280. A260/280 ratio and agarose gel electrophoresis were used to analyze the purity and quality of RNA. Reverse transcription was performed with 1μg total RNA according to the protocol suggested by the manufacturer (ABclonal). Complementary DNA was then used for real-time qPCR to determine the mRNA levels with Applied Biosystems 7900HT (ThermoFish). GAPDH was employed as an internal control. All primer sequences used in this study was listed in Supplementary Table S2.

**1.10 Chromatin immunoprecipitation assay**

Chromatin immunoprecipitation was performed as previously described[14]. H9c2 cells were cultured in 100mm dishes with 10ml complete culture medium. Paraformaldehyde was added directly into dishes at the final concentration of 1% and incubated at 37°C for 10 min to get cross-linked. By adding 1.1ml glycine solution (10X), cross-link was quickly stopped. Then, cells were washed with cold PBS and collected into centrifuge tubes. We used 0.2ml SDS Lysis Buffer with 1mM PMSF to suspend around one million H9c2 cells. Ultrasonic cell disruptor was employed to cut genomic DNA into 400 – 800bp fragment. After the preparation of ChIP samples, ChIP dilution buffer with 1mM PMSF was added to a final volume of 2ml. For precipitation, 20μl samples were used as input control. Almost 2ml samples were precleared with 70μl protein A/G agarose at 4°C for 1h to avoid unspecific binding. Cleared samples were then incubated with 1μg antibody to YB-1/Histone 3 or irrelevant IgG antibody at 4°C overnight. 60μl protein A/G agarose beads were used to precipitate the complex. Complex was washed with low salt immune complex wash buffer, high salt immune complex wash buffer, LiCl immune complex wash buffer and TE buffer carefully and separated at elution buffer. We used 20μl 5M NaCl to separate the protein and genomic DNA cross-linking and purified it with an assay kit. ChIP samples were stored at -80°C or used for real time qPCR immediately. The presence of immunoprecipitated DNA sequence around −1053/−1045 was detected by quantitative PCR. Primer sequences used in ChIP was listed in Supplementary Table S3.

**1.11 Dual luciferase assay**

To determine the effect of YB-1 expression on MEF2B transcriptional regulation, 293T cells were transfected with various doses of YB-1 overexpression plasmid, 10ng pRL-TK plasmid (Promega, Madison, WI), along with 400ng pGL3‐MEF2B-promoter reporter plasmids[15]. Next, to identify the effect of YB-1 on MEF2B transcriptional regulation under high glucose stimulation, YB-1 overexpression plasmid or YB-1 specific shRNA plasmid was employed in transfection system described above. To explore the binding site of YB-1 and MEF2B promoter, pGL3-basic, pGL3 (-2,022/+59) reporter plasmids, pGL3 (-1,393/+59) reporter plasmids, pGL3 (-919/+59) reporter plasmids, pGL3 (-440/+59) reporter plasmids or mutant pGL3‐MEF2B-promoter reporter plasmids were used. Culture medium was refreshed 6 h post transfection. Cells were washed by cold PBS for 3 times and added 50μl passive lysis buffer each well. After 3 times repeated freezing and thawing, samples were used to detect the luciferase activity by Dual-Luciferase Reporter Assay System (Promega) according to the manufacturer’s protocol.

**1.12 Reporter plasmid construction**

Around 2,000bp MEF2B promoter sequences was synthesized and cloned into pGL3-basic reporter plasmid by Sangon Biotech. Various fragments of MEF2B promoter sequences were amplified by PCR using the primers listed in Table S4. Fragments were cloned into pGL3-basic reporter plasmid by using Xhol and Hind III endonuclease and T4 DNA ligase (NEB). Mutation of the binding sites of YB-1 and MEF2B promoter was constructed using the primers listed in Table S5 with the Quick Mutation Site-Directed Mutagenesis Kit (Beyotime).

**References**

1. Madhusudhan T, Wang H, Dong W, Ghosh S, Bock F, Thangapandi VR, Ranjan S, Wolter J, Kohli S, Shahzad K, Heidel F, Krueger M, Schwenger V, Moeller MJ, Kalinski T, Reiser J, Chavakis T, Isermann B: Defective podocyte insulin signalling through p85-XBP1 promotes ATF6-dependent maladaptive ER-stress response in diabetic nephropathy. NAT COMMUN 2015;6:6496.

2. Wang T, Wu J, Dong W, Wang M, Zhong X, Zhang W, Dai L, Xie Y, Liu Y, He X, Liu W, Madhusudhan T, Zeng H, Wang H: The MEK inhibitor U0126 ameliorates diabetic cardiomyopathy by restricting XBP1's phosphorylation dependent SUMOylation. INT J BIOL SCI 2021;17:2984-2999.

3. Dai B, Li H, Fan J, Zhao Y, Yin Z, Nie X, Wang DW, Chen C: MiR-21 protected against diabetic cardiomyopathy induced diastolic dysfunction by targeting gelsolin. CARDIOVASC DIABETOL 2018;17

4. Huang Y, Long Y, Deng D, Liu Z, Liang H, Sun N, Xu Y, Lai Y, Cheng P: Alterations of anticoagulant proteins and soluble endothelial protein C receptor in thalassemia patients of Chinese origin. THROMB RES 2018;172:61-66.

5. Xue M, Dervish S, McKelvey KJ, March L, Wang F, Little CB, Jackson CJ: Activated protein C targets immune cells and rheumatoid synovial fibroblasts to prevent inflammatory arthritis in mice. Rheumatology (Oxford, England) 2019;58:1850-1860.

6. Zhou L, Miao K, Yin B, Li H, Fan J, Zhu Y, Ba H, Zhang Z, Chen F, Wang J, Zhao C, Li Z, Wang DW: Cardioprotective role of Myeloid-Derived suppressor cells in heart failure. CIRCULATION 2018;138:181-197.

7. Wang Y, Luo W, Han J, Khan ZA, Fang Q, Jin Y, Chen X, Zhang Y, Wang M, Qian J, Huang W, Lum H, Wu G, Liang G: MD2 activation by direct AGE interaction drives inflammatory diabetic cardiomyopathy. NAT COMMUN 2020;11:2148.

8. Ding M, Feng N, Tang D, Feng J, Li Z, Jia M, Liu Z, Gu X, Wang Y, Fu F, Pei J: Melatonin prevents Drp1-mediated mitochondrial fission in diabetic hearts through SIRT1-PGC1α pathway. J PINEAL RES 2018;65:e12491.

9. Ranjan S, Goihl A, Kohli S, Gadi I, Pierau M, Shahzad K, Gupta D, Bock F, Wang H, Shaikh H, Kähne T, Reinhold D, Bank U, Zenclussen AC, Niemz J, Schnöder TM, Brunner-Weinzierl M, Fischer T, Kalinski T, Schraven B, Luft T, Huehn J, Naumann M, Heidel FH, Isermann B: Activated protein C protects from GvHD via PAR2/PAR3 signalling in regulatory T-cells. NAT COMMUN 2017;8:311-316.

10. Zhang L, Chen J, Ning D, Liu Q, Wang C, Zhang Z, Chu L, Yu C, Liang H, Zhang B, Chen X: FBXO22 promotes the development of hepatocellular carcinoma by regulating the ubiquitination and degradation of p21. J EXP CLIN CANC RES 2019;38

11. Dong W, Wang H, Shahzad K, Bock F, Al-Dabet MM, Ranjan S, Wolter J, Kohli S, Hoffmann J, Dhople VM, Zhu C, Lindquist JA, Esmon CT, Grone E, Grone H, Madhusudhan T, Mertens PR, Schluter D, Isermann B: Activated protein c ameliorates renal Ischemia-Reperfusion injury by restricting Y-Box binding protein-1 ubiquitination. Journal of the American Society of Nephrology : JASN 2015;26:2789-2799.

12. Bao Z, Chen L, Guo S: Knockdown of SLC34A2 inhibits cell proliferation, metastasis, and elevates chemosensitivity in glioma. J CELL BIOCHEM 2019;120:10205-10214.

13. Li H, Dai B, Fan J, Chen C, Nie X, Yin Z, Zhao Y, Zhang X, Wang DW: The Different Roles of miRNA-92a-2-5p and let-7b-5p in Mitochondrial Translation in db/db Mice. Molecular Therapy - Nucleic Acids 2019;17:424-435.

14. Fang Q, Tian M, Wang F, Zhang Z, Du T, Wang W, Yang Y, Li X, Chen G, Xiao L, Wei H, Wang Y, Chen C, Wang DW: Amlodipine induces vasodilation via Akt2/Sp1‐activated miR‐21 in smooth muscle cells. BRIT J PHARMACOL 2019

15. Zhang X, Yuan S, Li H, Zhan J, Wang F, Fan J, Nie X, Wang Y, Wen Z, Chen Y, Chen C, Wang DW: The double face of miR-320: Cardiomyocytes-derived miR-320 deteriorated while fibroblasts-derived miR-320 protected against heart failure induced by transverse aortic constriction. Signal transduction and targeted therapy 2021;6:69.

**2. Supplementary Figures**

**Figure S1**

**
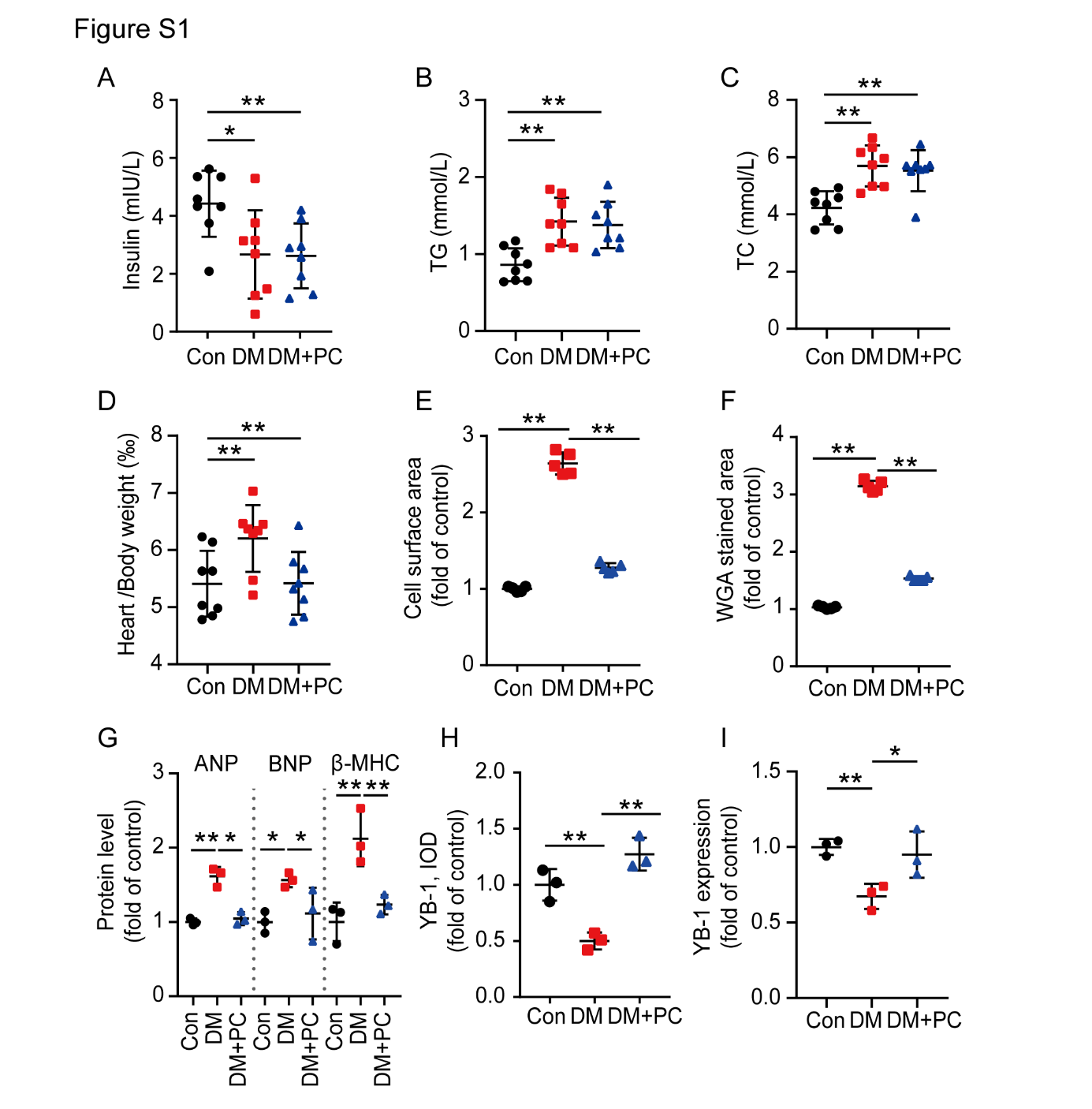
**

Figure S1: Plasma insulin (A), triglyceride (B) and total cholesterol level (C) were measured by commercially available ELISA kits. (D) Ratio of mouse heart to body weight measured at 34 weeks. For A-D, n=8 for all groups. (E) Scatter plot results of hematoxylin-eosin staining and (F) WGA staining illustrated the heart cell surface area of mice. Data were represented as fold of control, mean ± SD. For E-F, n=5 for all groups. (G) Scatter plot results of ANP, BNP, β-MHC protein abundance in heart tissues from diabetic mice treated with PC or not, versus controls. Total proteins were normalized to GAPDH. (H, I) Scatter plot summarizing IHC and western blot results demonstrating YB-1 protein level. Panels G - I, n=3 for all groups. Data were represented as fold of control, mean ± SD. Con, control; DM, diabetes mellitus; DM+PC, diabetes mellitus with PC treatment. **P*<0.05, ***P*<0.01. Student’s *t* test or one-way ANOVA, Bonferroni comparison test.

**Figure S2**


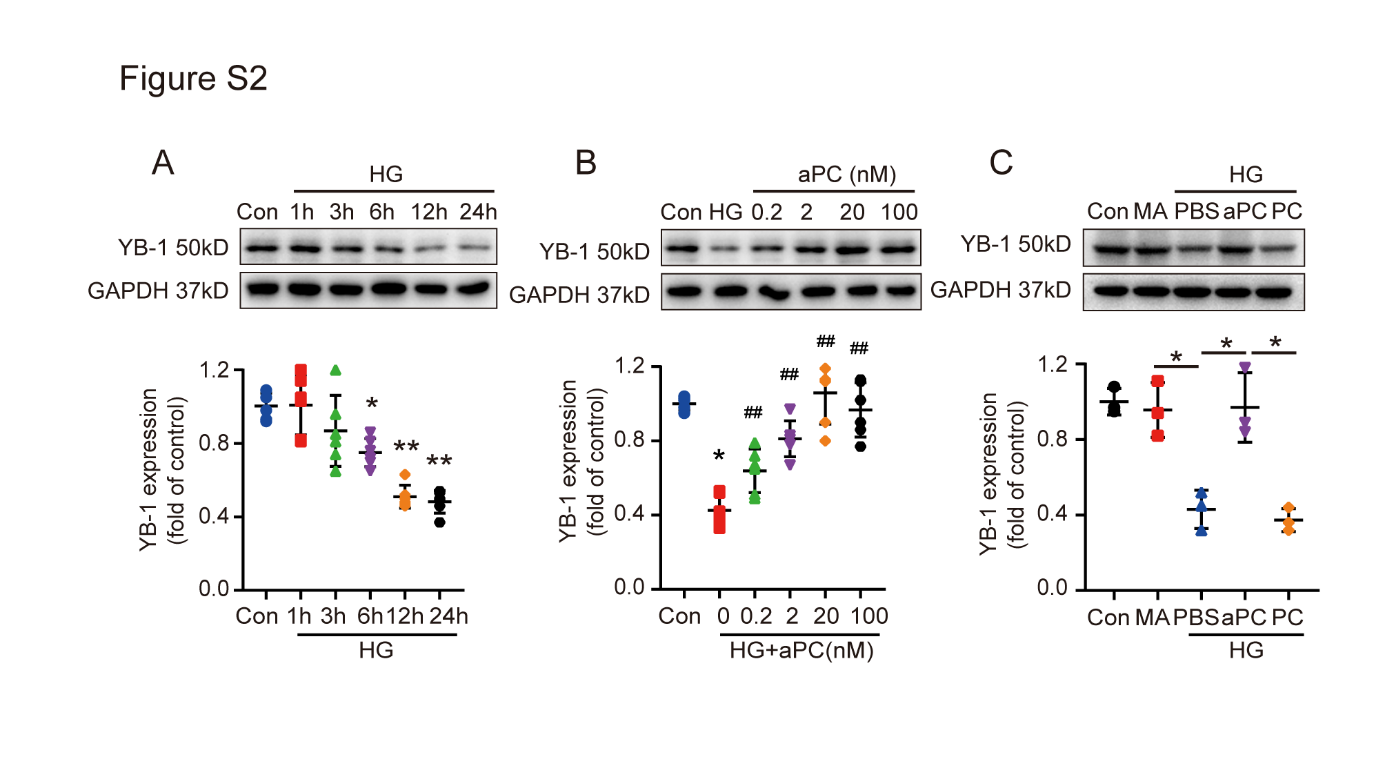


**Figure S2:** (A) YB-1 protein expression was detected in H9c2 cells challenged with HG (25mM) at indicated time points. (B) aPC maintained the YB-1 protein level under HG condition in a dose dependent manner. (C) Representative YB-1 western blots in H9c2 cells challenged by HG with aPC or PC pretreatment, mannitol acted as an osmotic control. Data were represented as fold of control, mean ± SD. for S2A-S2B, n=6 for all groups; for S2C, n=3 for all groups. Con, control; MA, mannitol; HG, 25mmol/L D(+) glucose; aPC, activated protein C; PC, protein C. **P*<0.05, ***P*<0.01, compared with control. *^##^ P*<0.01, compared with HG. Student’s *t* test or one-way ANOVA, Bonferroni comparison test.

**Figure S3**

**
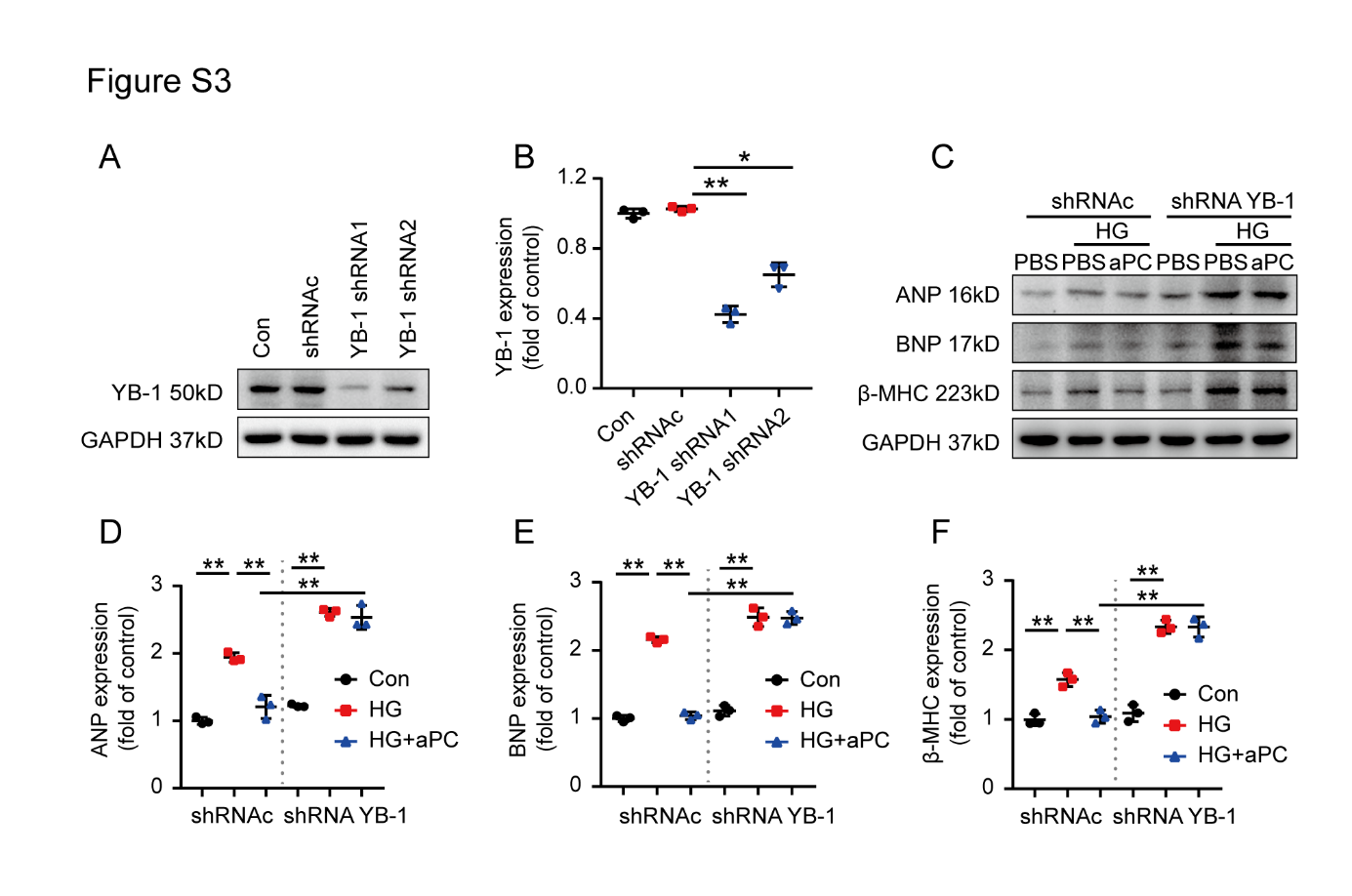
**

**Figure S3:** (A - B) Immunoblots of YB-1 in non-transfected H9c2 cells and H9c2 cells stably transfected with non-specific control shRNA (shRNAc) or different YB-1 specific shRNA (shRNA YB-1). GAPDH as loading control. Data were represented as fold of non-transfected H9c2 cells, mean ± SD. YB-1 shRNA1 showed a remarkable effect on suppressing YB-1 expression and was chosen for further experiment. n=3 for each group. (C) H9c2 cells were infected with control shRNA lentivirus or YB-1 shRNA lentivirus, co-incubated or not with HG (25mM, 6 h), pretreated or not with aPC (20nM, 30 min). Protein levels of ANP, BNP, β-MHC were determined by western blot. (D - F) Analyzed results of ANP, BNP, β-MHC protein abundance in mouse heart tissues. n=3 for each group. Con, control; HG, 25mM D(+) glucose; aPC, activated protein C. shRNAc, scrambled non-specific shRNA; shRNA YB-1, YB-1 specific shRNA. **P*<0.05, ***P*<0.01. Student’s *t* test or two-way ANOVA, Bonferroni comparison test.

**Figure S4**

**
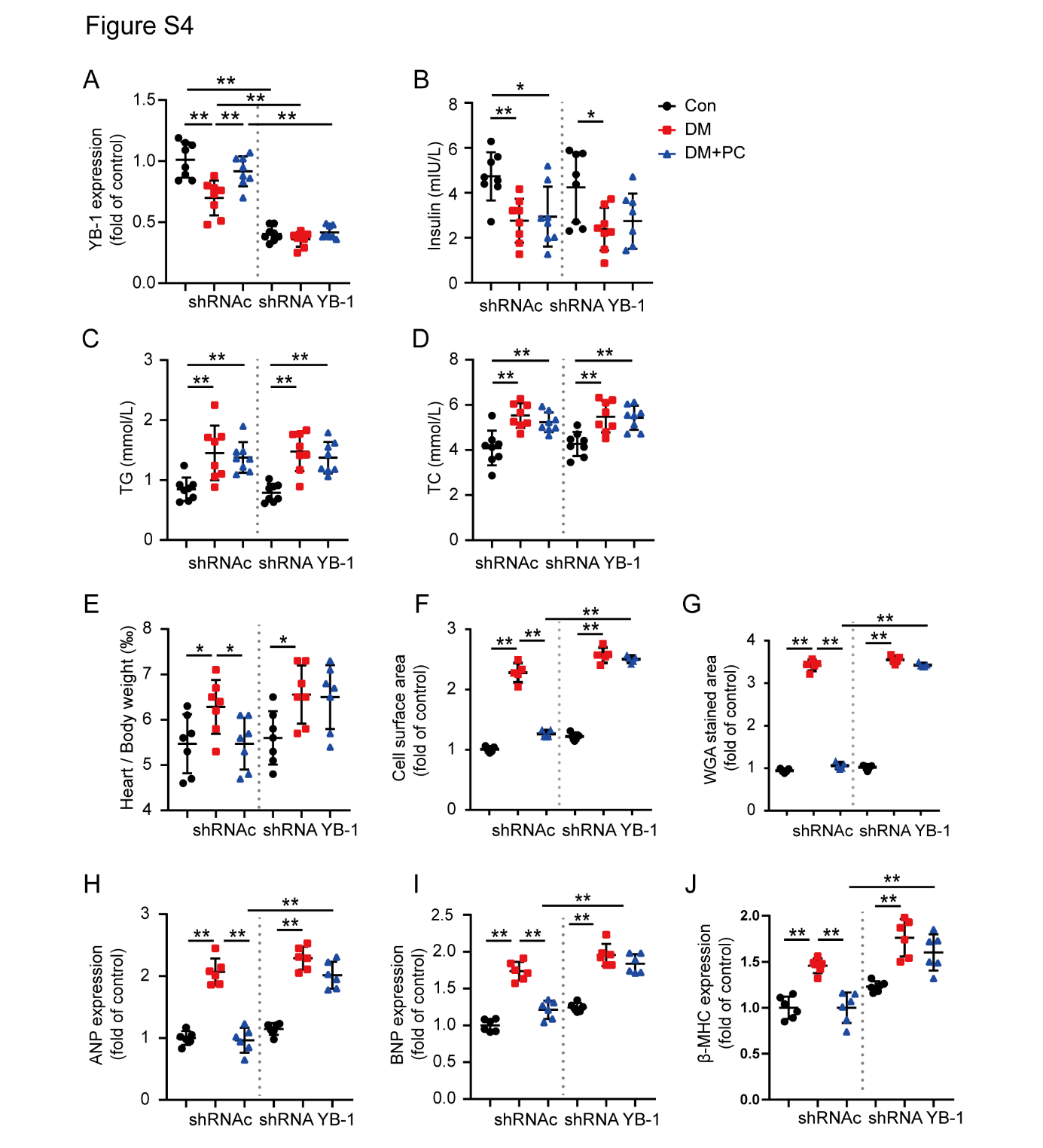
**

**Figure S4:** (A) Analysed results showing YB-1 knock down effect *in vivo*. Plasma insulin (B), triglyceride (C) and total cholesterol level (D) were measured by commercially available ELISA kits. (E) Ratio of mouse heart weight to body weight. (F, G) Cell surface area of mouse heart tissue conformed by hematoxylin-eosin staining and WGA staining in YB-1 knock down mice or counterparts. (H - J) Scatter plot results of protein abundance of ANP, BNP, β-MHC. Data were presented as mean ± SD. n≥5 for all groups. Con, control; DM, diabetes mellitus; DM+PC, diabetes mellitus with PC treatment. shRNAc, scrambled non-specific shRNA; shRNA YB-1, YB-1 specific shRNA. **P*<0.05, ***P*<0.01. Student’s *t* test or two-way ANOVA, Bonferroni comparison test.

**Figure S5**

**
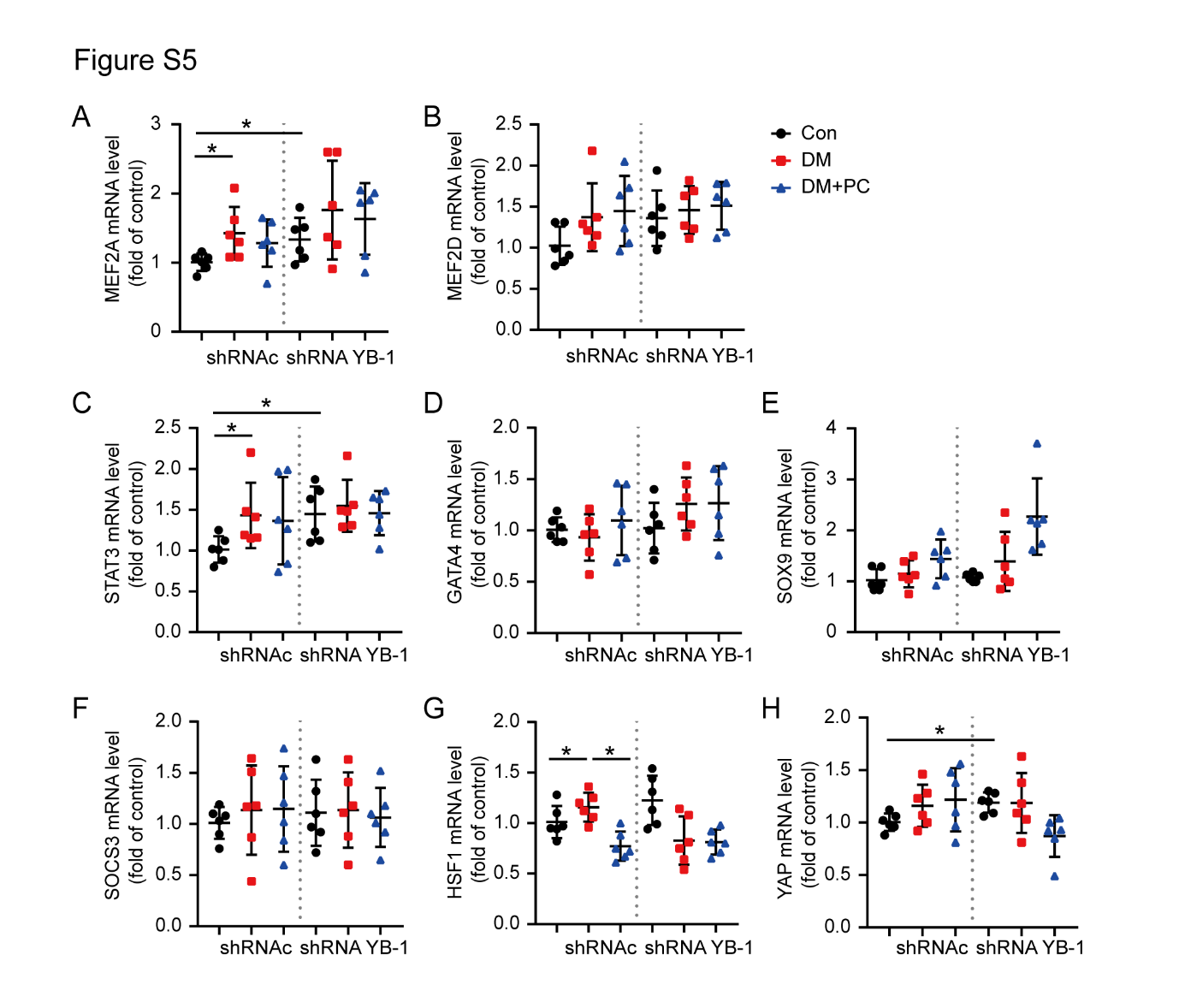
**

**Figure S5:** (A - H) mRNA levels of different transcription factors related to heart failure in mouse heart tissues. Data were represented as fold of control, mean ± SD, n=6 for all groups. **P*<0.05. Two-way ANOVA, Bonferroni comparison test. Each sample was measured in triplicate.

**Figure S6**

**
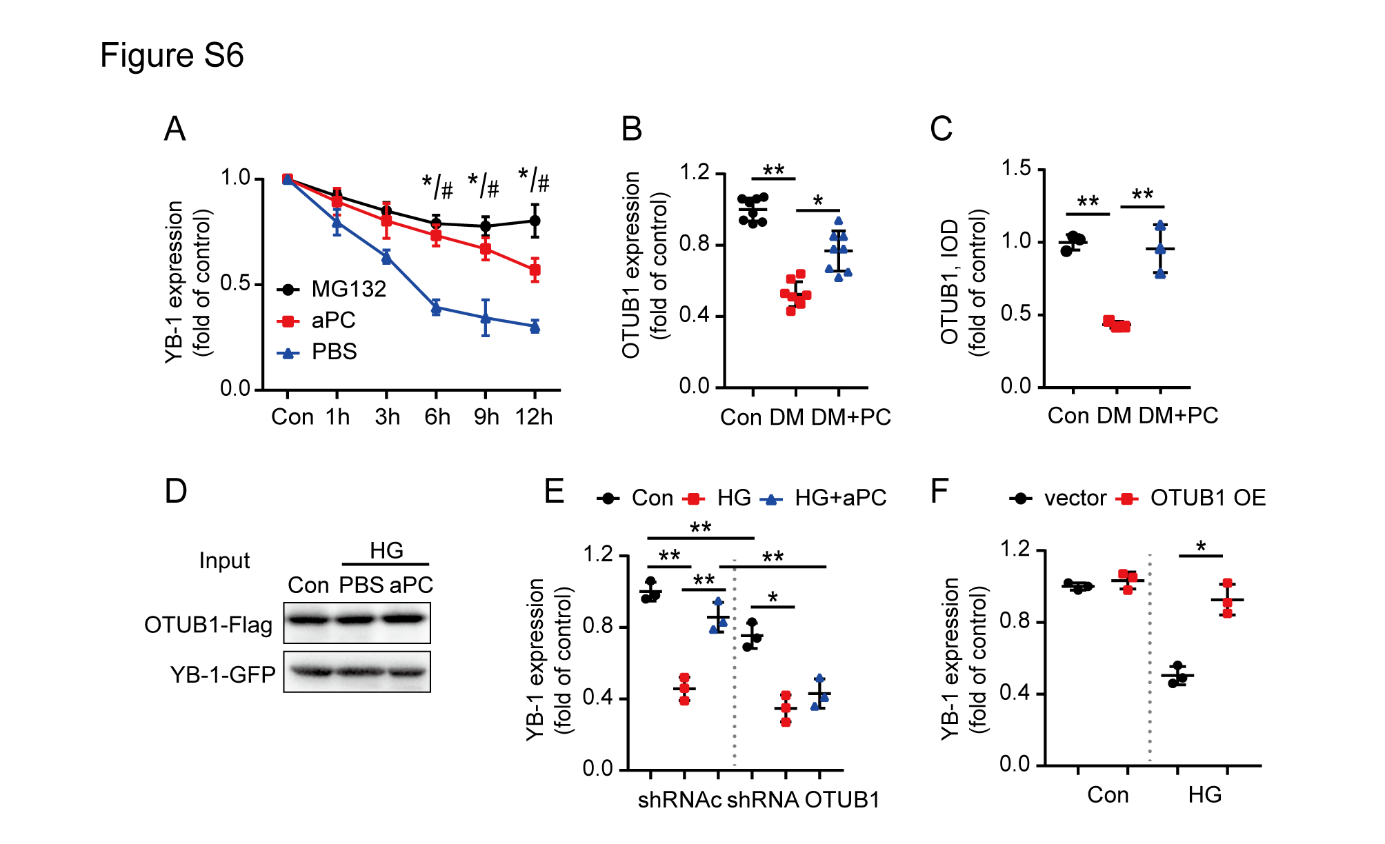
**

**Figure S6:** (A) Analyzed results showing the effect of preconditioning of MG132, aPC or PBS on degradation of YB-1 protein. (B-C) Scatter plot results of western blots or immunohistochemistry staining showing OTUB1 expression in mouse heart tissue. (D) Representative blots of input control for Figure 4G. H9c2 cells were infected with both GFP-labelled YB-1 overexpression adenovirus and Flag-labelled OTUB1 overexpression adenovirus. (E) Scatter plot results of YB-1 expression in control and OTUB1 knock down H9c2 cells. (F) Scatter plot results of YB-1 expression in control and OTUB1 overexpression H9c2 cells. Data were represented as fold of control, mean ± SD. for panel B, n=8 for each group; for other panels, n=3 for all groups. Con, control; HG, 25mmol/L D(+) glucose; aPC, activated protein C; PC, protein C; shRNAc, control non-specific shRNA; shRNA OTUB1, OTUB1 specific shRNA; CHX, cycloheximide; DM, diabetes mellitus; DM+PC, diabetes mellitus with PC treatment; OE, overexpression. **P*<0.05, ***P*<0.01. Student’s *t* test or one-way ANOVA, Bonferroni comparison test.

**Figure S7**

**
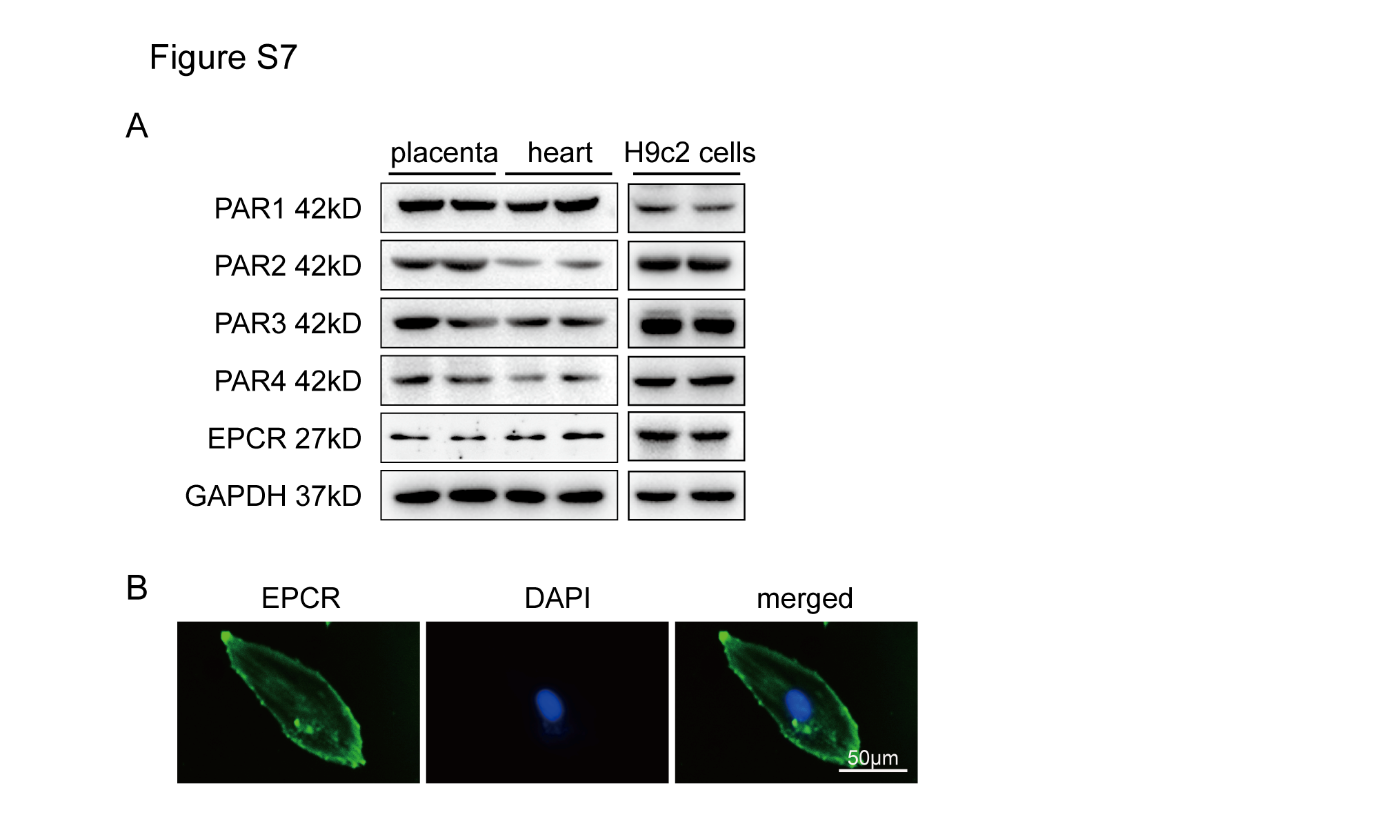
**

**Figure S7:** (A) Representative western blots showing expression of PARs and EPCR in mouse placenta, heart tissue lysates and H9c2 cardiomyocytes. Placenta served as positive control. (B) Representative immunofluorescent staining pictures of EPCR (green) in cultured cardiomyocyte, nucleus is counterstained with DAPI (blue). Scale bar, 50μm.

**3. Supplementary Tables**

**Table S1: PARs agonist and control peptides sequences**

| Category | Abbr. | Sequence |
| --- | --- | --- |
| PAR1 agonist peptide | P1 AP | TFLLR |
| PAR2 agonist peptide | P2 AP | SLIGRL |
| PAR3 agonist peptide | P3 AP | SFNGGP |
| PAR4 agonist peptide | P4 AP | AYPGKF |
| control peptide | Con P | YAPGKF |

**Table S2: Primer sequences used for real time quantitive PCR to detect relative mRNA levels in mouse heart tissue.**

| Gene | Forward | Reverse |
| --- | --- | --- |
| MEF2A | ATTCCCCCATCAAGCAAGGG | TGTTGTAGGCTGTCGGCATT |
| MEF2B | TGGGACACCCGATCTTCTCT | GCTGTACAGGCTTCTCAGGC |
| MEF2D | CCCGTTTCTCTCAGCAACCT | CTTGATGCTGATGTGGGGGT |
| YAP1 | CCCTCGTTTTGCCATGAACC | GCTGTATTTGCTGCTGCTGG |
| SOCS3 | TGC​GCC​TCA​AGA​CCTTCA​G | GCTCCA​GTA​GAA​TCC​GCTCTC |
| HSF1 | GCCCCTCTTCCTTTCTGCAT | TCATGTCGGGCATGGTCAC |
| STAT3 | CGAAGCCGACCCAGGTAGT | GCATCAATGAATGGTGTCACACAG |
| GATA4 | CTGTGCCAACTGCCAGACTA | TTTGAATCCCCTCCTTCCGC |
| SOX9 | GGCTCCTACTACAGTCACGC | AGACTGGTTGTTCCCAGTGC |
| GAPDH | CCCTTAAGAGGGATGCTGCC | TACGGCCAAATCCGTTCACA |

**Table S3: Primer sequences used for ChIP.**

| Gene | Forward | Reverse |
| --- | --- | --- |
| MEF2B promoter 1 | ACAGCCCTTGCCCCAATT | ATCCATGCACTCCCCTAACC |
| MEF2B promoter 2 | TTGAAAGCCCAGCACAAATG | TGCACTCCCCTAACCCTGTG |

**Table S4: Primer sequences used for various MEF2B promoter segments luciferase repoter plasmid construction.**

| Name | Sequence |
| --- | --- |
| MEF2B +59-R | AAGCTTCTGCTCGGCCTGGGCC |
| MEF2B -440-F | CTCGAGAGCGTTGCCTGCGAGAAG |
| MEF2B -919-F | CTCGAGAGGGTTAGGGGAGTGCATGG |
| MEF2B -1393-F | CTCGAGGAAAAAGCCCTTCTCGGGTC |
| MEF2B -2012-F | CTCGAGGTAACCAGAGCCTGCCTGTTG |

**Table S5: Primer sequences used for mutant MEF2B promoter repoter plasmid construction.**

| MEF2B-mut-F | CTGTCCCAGGCACGCTTGCCTCCTCTGGCCCAAGCCTGAC |
| --- | --- |
| MEF2B-mut-R | GACAGGGTCCGTGCGAACGGAGGAGACCGGGTTCGGACTG |
